# Recovery of synaptic loss and depressive-like behavior induced by GATA1 through blocking of the neuroinflammatory response

**DOI:** 10.3389/fncel.2024.1369951

**Published:** 2024-05-09

**Authors:** Koeul Choi, Joonhee Lee, Gukdo Kim, Younghyun Lim, Hyo Jung Kang

**Affiliations:** Department of Life Science, Chung-Ang University, Seoul, Republic of Korea

**Keywords:** depression, GATA1, multi-omics, microglia, inflammation

## Abstract

GATA1, a member of the GATA transcription factor family, is a critical factor in hematopoietic system development. In a previous study, we demonstrated the increased expression of GATA1 in the dorsolateral prefrontal cortex (dlPFC) of patients suffering from depression and described its role as a transcriptional repressor of synapse-related genes. In this study, we investigated how GATA1 globally altered gene expression using multi-omics approaches. Through the combined analyses of ChIPseq, mRNAseq, and small RNAseq, we profiled genes that are potentially affected by GATA1 in cultured cortical neurons, and Gene Ontology (GO) analysis revealed that GATA1 might be associated with immune-related functions. We hypothesized that GATA1 induces immune activation, which has detrimental effects including synapse loss and depressive-like behavior. To test this hypothesis, we first performed a microglial morphometric analysis of a brain having overexpression of GATA1 because microglia are the resident immune cells of the central nervous system. Fractal analysis showed that the ramification and process length of microglia decreased in brains having GATA1 overexpression compared to the control, suggesting that GATA1 overexpression increases the activation of microglia. Through flow cytometry and immunohistochemical analysis, we found that activated microglia showed pro-inflammatory phenotypes characterized by the expression of CD86 and CD68. Finally, we demonstrated that the effects of GATA1 overexpression including synapse loss and depressive-like behavior could be blocked by inhibiting microglial activation using minocycline. These results will elucidate the regulatory mechanisms of GATA1 that affect pathophysiological conditions such as depression and provide a potential target for the treatment of depression.

## 1 Introduction

Major depressive disorder (MDD) is a highly prevalent and devastating psychiatric disorder that affects millions of people worldwide (James et al., 2018). Despite growing awareness of its impact, the underlying mechanisms of MDD remain poorly understood (Kharade et al., 2010; Cai et al., 2015). Inflammation has emerged as a potential factor in the pathophysiology of depression, and neuroinflammation has been suggested as a key component in the development of MDD (Miller and Raison, 2016). Previously, the central nervous system (CNS) has been considered as an immune-privileged site because of the presence of blood–brain barrier (BBB; Quan and Banks, 2007). However, it is currently understood that the CNS communicates with the immune system via several routes and that neuroinflammation, defined as the inflammatory response of nervous tissues such as the brain or spinal cord, is a potential mechanism underlying the pathophysiology of depression (Wrona, 2006; Dantzer, 2018).

GATA1 is a member of the GATA transcription factor family, characterized by the two zinc-finger domains that bind to the DNA consensus sequence (A/T) GATA (A/G; Ko and Engel, 1993). The GATA family is divided into two subfamilies based on their expression profiles (Burch, 2005). GATA1, along with GATA2 and GATA3, belongs to the hematopoietic subfamily and is expressed in hematopoietic lineage cells such as erythrocytes, megakaryocytes, mast cells, eosinophils, basophils, and dendritic cells (Gao et al., 2015). The knockout of GATA1 in mice showed embryonic lethality caused by severe anemia, which indicates that GATA1 is an essential factor for the development and maturation of hematopoietic lineage cells (Ferreira et al., 2005). Accordingly, GATA1 has been linked to several diseases including cytopenias and acute megakaryoblastic leukemia (Ciovacco et al., 2008). These blood disorders are caused by inherited mutation and somatic mutation that alter the ability of GATA1 to bind to the DNA sequence and produce the short form of the GATA1 protein (Crispino, 2005). Increased levels of GATA1 have also been associated with several blood diseases (Whyatt et al., 2000; Caldwell et al., 2013; Lally et al., 2019). In addition to its functions in the hematopoietic system, GATA1 has a role as a transcription regulator in the CNS. GATA1 shows increased expression in the dorsolateral prefrontal cortex (dlPFC) of patients with MDD and is also induced by chronic unpredictable stress in the PFC of rat brains (Kang et al., 2012). Recent evidence suggests that GATA1 may also play a role in activating the immune response in the CNS, potentially contributing to neuroinflammation (Choi et al., 2016).

In this study, we investigated the global alteration in gene expression by GATA1 in cultured cortical neurons using ChIPseq, mRNAseq, and small RNAseq. Our analysis reveals that GATA1 is involved in a variety of biological processes, including immune-related functions. We hypothesized that GATA1 induces immune activation, which may lead to synapse loss and depressive-like behavior. To test this hypothesis, we performed a microglial morphometric analysis and found that GATA1 overexpression increased the activation of microglia, resulting in a pro-inflammatory phenotype. Finally, we demonstrated that the effects of GATA1 overexpression, including synapse loss and depressive-like behavior, could be blocked by inhibiting microglial activation using minocycline. These findings may provide insights into the regulatory mechanisms of GATA1 that affect the pathophysiology of depression and suggest potential targets for future therapies.

## 2 Materials and methods

### 2.1 Primary cortical neuron culture

Embryonic brains were prepared from ICR mice after 14 d of gestation. Embryonic cortices were dissected and incubated at 37°C for 15 min in 1.54 units of papain (Worthington Biochemical Corp., Lakewood, NJ, United States, Cat# LK003176) followed by trituration using a glass fire-polished pasture pipette. Dissociated cells were plated on 6-well plates (1.2 × 10^6^ cells per well) coated with poly-L-Lysine in a culture medium consisting of neurobasal media supplemented with 10% FBS, 2 mM L-glutamine, 1 mM sodium pyruvate, 1 mM HEPES, 2% Pen-Strep, and 2% B27 supplement. The medium was changed after 1 day *in vitro* (DIV) to halt proliferation of non-neuronal cells. After 8 h, the neurons were infected with AAV-Control or GATA1. Cultures were then maintained at 37°C in a humidified 5% CO_2_ atmosphere.

### 2.2 Chromatin immunoprecipitation

Cultured cortical neurons (at DIV11) were cross-linked with 1% (v/v) formaldehyde, followed by adding 125 mM of glycine to quench the cross-linking reaction. The cells were lysed in SDS lysis buffer (1% SDS, 1 mM EDTA, and 50 mM Tris–HCl, pH 8.0) including protease inhibitors and phosphatase inhibitors. Lysate was sonicated to produce chromatin fragments of 200–500 bp. Sheared chromatin was immunoprecipitated with Dynabead (Thermo Fisher Scientific, Inc., Waltham, MA, United States, Cat# 10003D), conjugated with 1 ug of H3K4me3 (Abcam, Cambridge, United Kingdom, Cat# ab8580), H3K27me3 (Merck, Rahway, NJ, United States, Cat# 07–449)-specific antibodies, or control IgG antibodies (Cell Signaling Technology, Inc., Danvers, MA, United States, Cat# 2729) overnight at 4°C with rotation. After reverse crosslinking, DNA was purified by using phenol-chloroform and ethanol precipitation.

### 2.3 ChIP sequencing

The ChIPed DNA was used for library preparation using NEBNext^®^ Ultra^™^ DNA library Prep Kit for Illumina^®^ (New England Biolabs, Ipswich, MA, United States) as per the manufacturer’s instructions. The ChIPed DNA was ligated with adaptors. After purification, a PCR reaction was carried out with adaptor-ligated DNA and index primer for multiplexing sequencing. The library was purified by using magnetic beads and sequenced on a HiSeq2500 system as 75 bp paired ends (Illumina, San Diego, CA, United States). Sequence reads were mapped to the mouse genome (mm9) using BWA (Li and Durbin, 2009). Peak calling was performed using the HOMER findPeaks program,^1^ input DNA without ChIP as reference, using the default settings except for region size, which was 75, and minDist, which was 200. Peaks were annotated using HOMER annotatePeaks.pl. tool.^2^ The ChIPseq peaks were visualized using Integrative Genomics Viewer (IGV, v2.8.0).

### 2.4 Total RNA extraction

Total RNA extraction was performed using an miRNeasy mini kit (Qiagen, Hilden, Germany, Cat# 217004). At DIV 11, the culture neurons were washed twice using cold phosphate-buffered saline (PBS) and added directly to 700 μL of QIAzol lysis reagent. The lysates were vortexed for 1 min, combined with chloroform, and centrifuged for 15 min at 12000 g at 4°C. The supernatant was combined with 100% ethanol and applied to the spin column. Before elution, DNase I treatment (Qiagen, Cat# 79524) was conducted directly on the spin column. Subsequent processes were performed according to the manufacturer’s instructions. RNA concentration was quantified using a NanoDrop Spectrophotometer (Thermo Fisher Scientific, Inc.) and Agilent 2,100 BioAnalyzer (Agilent, Santa Clara, CA, United States).

### 2.5 mRNA sequencing

The libraries were prepared for 100 bp paired-end sequencing using TruSeq Stranded mRNA library preparation kit (Illumina). The fragmented mRNAs were synthesized as single-stranded cDNAs through random hexamer priming. By applying this as a template for second strand synthesis, double-stranded cDNA was prepared. The cDNA libraries were amplified with PCR and sequenced as paired-end (2 × 100 bp) using Illumina HiSeq2500 platform (Illumina). Low-quality reads containing more than 10% skipped bases, more than 40% bases whose quality scores are less than 20, or wherein the average quality score of each read is less than 20 were filtered. Filtered reads were mapped to the mouse genome (mm10) using the aligner TopHat (v2.1.1; Trapnell et al., 2009). The data were assembled into transcripts using Cufflinks (v2.1.1; Trapnell et al., 2010). Differential expression analysis was performed using Cuffdiff (v2.1.1; Trapnell et al., 2012). To enhance the analysis accuracy, multi-read correction and frag-bias-correct options were applied. All other options were set to default values. The *p*-values were corrected through the Benjamini–Hochberg method. Genes with raw *p*-value <0.05 were considered to be differentially expressed.

### 2.6 Small RNA sequencing

Small RNA sequencing libraries were constructed using NEXTFLEX^®^ Small RNA-seq Kit v3 for Illumina^®^ Platform (PerkinElmer, Waltham, MA, United States). The adapters are directly, and specifically, ligated to the microRNA molecules. The 5′ and 3’ NEXTFLEX adapter ligated products were reverse-transcribed to create single-stranded cDNA. The cDNA is then PCR amplified. The amplified cDNA constructs were separated on a 6% TBE gel, and the 140–160 bp bands were excised. After gel purification, the library was sequenced in 100PE mode using Illumina HiSeq2500 platform (Illumina). After removing the sequencing adapters, quality control checks on raw reads were performed using Fast QC v0.11.5.^3^ Low-quality bases at the 3′ ends (< Q20) were trimmed and reads shorter than 17 nucleotides were discarded. The remaining high-quality reads were mapped to the reference mouse genome (mm10) using Bowtie v1.1.2^4^ (Langmead et al., 2009) with the following parameters: only 1 mismatch (−n 1), max 80 mismatch quals across alignment, 30 seed length, and the suppression of reads showing >5 alignments. The number of reads that mapped to each gene in each sample were counted using HTseq v0.6.1p1 (Anders et al., 2015). Differentially expressed genes were identified based on the trimmed mean of M value (TMM) normalization method using edgeR v3.10.2 in R package, using the default parameters (Robinson et al., 2010). The *p*-values were calculated using Fisher’s exact test and corrected through the Benjamini–Hochberg method using the p.adjust function in R package. Genes with raw *p*-value <0.05 were considered to be differentially expressed.

### 2.7 Target prediction

The target genes of differentially expressed miRNAs were predicted using miRDB v5.0,^5^ miRanda (August 2010 release),^6^ DIANA tools v5.0,^7^ miRmap (mirmap201301e),^8^ and TargetScan v7.2.^9^ The top 50 genes were obtained from each database, and the genes presented in two or more databases were selected as potential target genes.

### 2.8 Gene ontology analysis

Functional enrichment of the genes was performed using Database for Annotation, Visualization and Integrated Discovery (DAVID).^10^ As the input limit is 3,000 genes, only the top 3,000 genes were used for the unique peak of the H3K27me3 promoter region based on peak score. For ChIPseq and small RNAseq, the significant categories were identified (Benjamini <0.1). For RNAseq, the significant categories were identified (*p*-value <0.05).

### 2.9 Animal

ICR mouse embryos and adult male C57BL/6 mice (8 weeks, 25–27 g) were used in this study. The animals were housed five per cage with *ad libitum* access to food and water in a 12-h light–dark cycle. Mice were kept in the laboratory animal room for at least 3 days before the behavior test began. All experiments were approved by the Committee on Institutional Animal Care and Use of Chung-Ang University (Seoul, Korea).

### 2.10 Virus

The AAV-control (pAAV_DJ_-EF1α-MCS-CMV-EGFP; 6,052 bp) vector was purchased from SBI (System Biosciences, LLC, Palo Alto, CA, United States, Cat# AAV536A-1). To generate AAV-GATA1, mouse GATA1 cDNA (1801 bp) was inserted into the AAV-control vector by enzyme restriction and ligation. The AAV was packaged in the Korea Institute of Science and Technology (KIST) virus facility.^11^ The titer of the AAV-control was 0.6 × 10^12^ GC/mL while that of AAV-GATA1 was 1.4 × 10^11^ GC/mL.

### 2.11 Stereotaxic surgery

Mice were anesthetized with Rompun (9.3 mg/kg) and ketamine (87.5 mg/kg). Bilateral viral injections were performed with coordinates +1.7 mm (anterior/posterior), +0.75 mm (lateral), and − 2.5 mm (dorsal/ventral) relative to the bregma. A total of 1 μL of purified virus was delivered at a rate of 0.1 μL/min. The needle was kept in this position for an additional 5 min after injection and then removed slowly from the brain. The scalp incision was then closed using sutures.

### 2.12 Tissue preparation and immunofluorescence

Mice were anesthetized and transcardially perfused with ice-cold PBS, followed by 4% paraformaldehyde (PFA) in PBS. After the cervical dislocations, the brains were immediately isolated and post-fixed overnight in 4% PFA and then immersed in 30% sucrose at 4°C. The brains were quickly frozen using chilled isopentane. Frozen tissues were cut into 20-μm sections on a cryostat (Leica Instruments, Wetzlar, Germany). Free-floating coronal sections were washed in PBS and then incubated in 0.3% hydrogen peroxide for 5 min, followed by proteinase K treatment for 10 min and incubation in normal horse serum for 10 min. After blocking with 1% BSA in PBS (0.2% Tween 20) for 1 h at room temperature, the sections were incubated with goat polyclonal anti-Iba1 (1:200, Abcam, Cat# ab5076) in PBS (0.2% Tween 20, 1% BSA) for 1 h at room temperature and then overnight at 4°C. For double staining, sections were first incubated with goat anti-Iba1 for 1 h at room temperature, followed by incubation with rabbit polyclonal anti-CD68 (1:100, Abcam, Cat# ab125212) for 1 h at room temperature and then overnight at 4°C. Subsequently, the sections were incubated with secondary antibodies (1:200, Abcam, Cat# ab150063, ab150136) for 1 h at room temperature. Stained sections were mounted on glass slides using a mounting medium containing DAPI (Vector Laboratories, Inc., Newark, CA, United States, Cat# H-1200).

### 2.13 Image processing and microglial morphometric analysis

For microglial morphometric analysis, fluorescent images were acquired using BioTek Lionheart FX (BioTek, Winooski, VT, United States) in z-stack mode (0.5 μm thick × 40 images) at 20× magnification. Acquired images of a color TIFF format were processed for further analysis using FIJI free software^12^ using the following steps: (i) conversion to 8-bit grayscale, (ii) adjustment of brightness and contrast, (iii) processing of images to make them unsharp using Gaussian blur, and (iv) transformation into a binary image using threshold function. Subsequently, individual microglial cells were randomly selected and cropped to the same size (340 × 340 pixels). Single-cell images were edited manually to eliminate noise. Finally, “filled images” and their counterpart “outlined images” were used for morphometric analysis. All parameters were measured with the FracLac for Image J using the box-counting method as previously described (Karperien et al., 2013; Fernandez-Arjona et al., 2017).

### 2.14 Hierarchical cluster analysis

Hierarchical cluster analysis was performed based on the measured morphometric parameters using R (v3.6.1). The number of appropriate clusters were determined using NbClust packages in R (Charrad et al., 2014). Euclidean distance was used to evaluate similarity between samples using Ward’s method (Ward Jr, 1963). Finally, the clustering was displayed in the form of a dendrogram.

### 2.15 Brain mononuclear cell isolation and flow cytometry

Mice were sacrificed 4 weeks after virus injection. For discrimination of circulating cells and cells residing within the tissue, circulating cells were labeled with anti-CD45.2 APC (104; Tonbo Biosciences, San Diego, CA, United States, Cat# 20–0454) for 3 min through intravenous injection. Mice were anesthetized and transcardially perfused with PBS. After cervical dislocations, the brains were removed and rinsed with ice-cold PBS. The whole brain was ground using a Dounce homogenizer, followed by enzymatic dissociation including collagenase D (2 mg/mL; Sigma-Aldrich, St. Louis, MO, United States, Cat# 11088858001) and DNase I (20 mg/mL; Sigma-Aldrich, Cat# 11284932001) for 30 min at 37°C. The homogenate was resuspended in RPMI with 10% FBS and centrifuged for 7 min at 300 g at 18°C. Cells were resuspended in 37% Percoll (Sigma-Aldrich, Cat# P1644) solution and centrifuged for 40 min at 200 g at 18°C without brakes. After myelin removal, cells were washed with FACS buffer consisting of 1x PBS with 2 mM EDTA, 1% BSA, and 0.1% sodium azide, followed by incubation with anti-CD45 PE (30-F11; Tonbo Biosciences, Cat# 50–0451), anti-CD11b PerCP-Cy5.5 (M1/70; Tonbo Biosciences, Cat# 65–0112), anti-CD86 APC-Cy7 (GL-1; Biolegend, San Diego, CA, United States, Cat# 105029), and anti-CD206 PE-Cy7 (MR6F3; eBioscience, San Diego, CA, United States, Cat# 25–2061-80) for 20 min at 4°C. After washing, the stained-positive cells were assayed using Attune NxT Flow cytometer (Thermo Fisher Scientific, Inc.). FlowJo v10 software was used for the data analysis that followed.

### 2.16 Image acquisition and quantification of CD68^+^ microglia

Dual-labeled fluorescent images were acquired using Eclipse Ti2-E equipped with an N-SIM unit (Nikon, Tokyo, Japan). To quantify the number of Iba1^+^CD68^+^ co-localized cells, images at 10x magnification with an intermediating magnification switch of 1.5x were captured randomly throughout the AAV-injected region expressing green fluorescence. After pseudo-color processing of CD68-labeled images, the Iba1-labeled image and CD68-labeled image were merged using Image J. The number of Iba1^+^ and Iba1^+^CD68^+^ co-localized cells were counted manually and then the percentage of CD68^+^ microglia was determined as per the following formula: number of Iba1^+^CD68^+^ cells/total number of Iba1^+^ cells. For high-magnification images, the images were captured with a 40x/0.95 NA objective with an intermediating magnification switch of 1.5x in 3D-SIM mode. Image stacks were collected with a z-step size of 0.5 μm and processed using NIS-Element AR (v5.30.02).

### 2.17 Quantitative real-time PCR

Mice were sacrificed 4 weeks after the virus injection. After cervical dislocations, the brains were removed and rinsed using ice-cold DEPC-PBS. The mPFC was dissected using the mouse brain atlas (Paxinos and Franklin 4th Edition) as a reference. The dissected tissues were homogenized in a Bullet Blender (Next Advance, Inc., Troy, NY, United States) using QiAzol lysis reagent (Qiagen) and RNase-free beads. Total RNA concentration was quantified using NanoDrop Spectrophotometer (Thermo Fisher Scientific, Inc.). Then, 500 ng of total RNA was used for cDNA synthesis using oligo dT primers and DiaStar RTase (SolGent, Daejeon, Korea, Cat# DR23-R10k). PCR reactions were conducted on an ABI QuantStudio 6 (Thermo Fisher Scientific, Inc.) using a SYBR-green-based method (power SYBR green PCR master mix, Thermo Fisher Scientific, Inc., Cat# 4367659). The Ct value (cycle number at threshold) was used for comparison of relative amount of mRNA molecules. All of the genes of interest were normalized to the housekeeping gene, *Gapdh*.

### 2.18 Minocycline administration

Mice, aged 8 weeks, were subjected to stereotaxic surgery for virus infusion and given a recovery duration of a week. They were divided into four groups, with 9–10 mice in each group: (Group 1) control + normal saline (CT_NS), wherein mice infused with AAV-control were injected with normal saline; (Group 2) control + minocycline (CT_MIN), wherein mice infused with AAV-control were injected with minocycline; (Group 3) GATA1 + normal saline (GT_NS), wherein mice infused with AAV-GATA1 were injected with normal saline; and (Group 4) GATA1 + minocycline (GT_MIN), wherein mice infused with AAV-GATA1 were injected with minocycline. Minocycline (Sigma-Aldrich, Cat# M9511) was administrated intraperitoneally daily for 21 days. The concentration of minocycline was 30 mg/kg and injection volumes were 10 mL/kg. Normal saline was administrated in the same volume.

**Figure 1:**
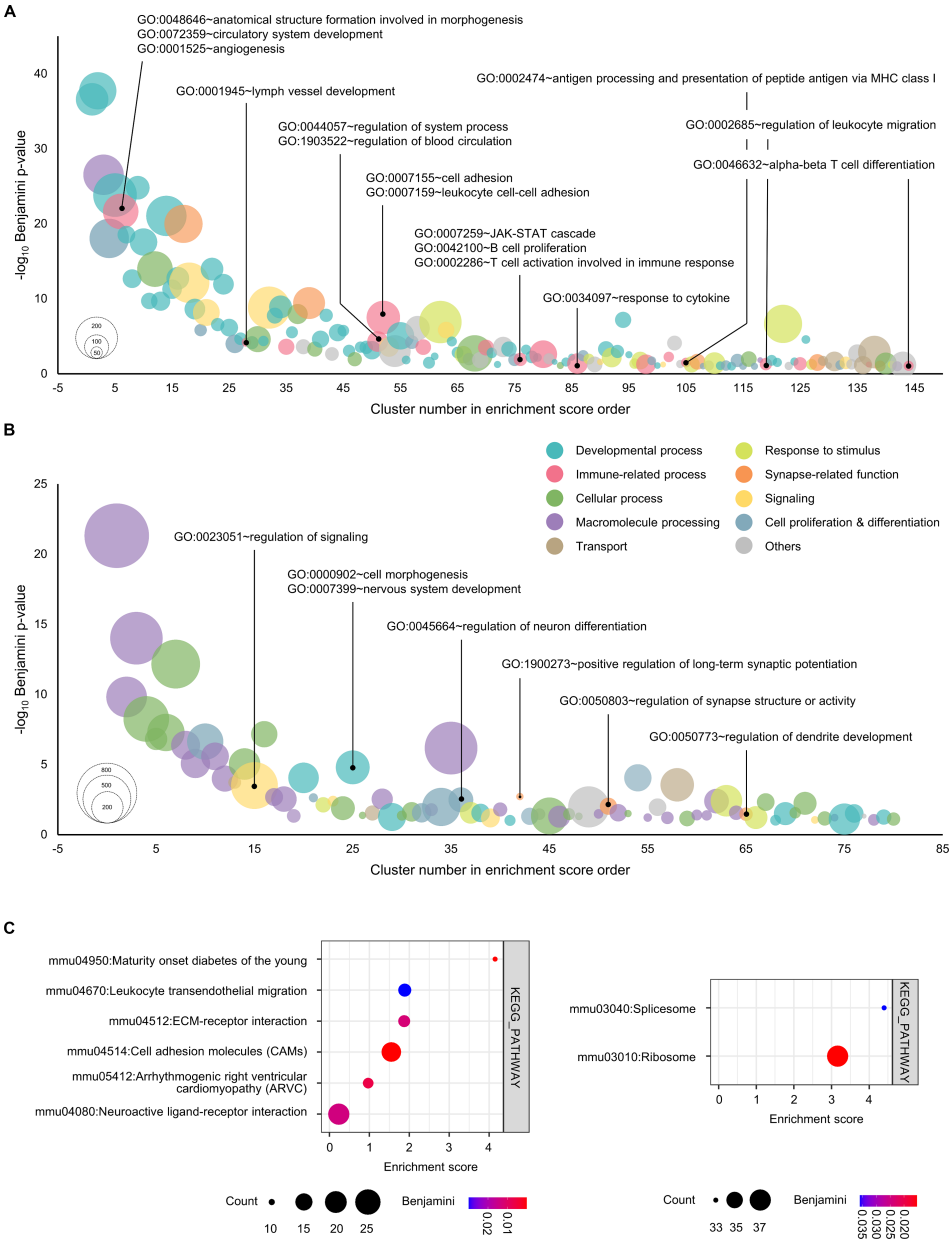
Functional annotation of genes for unique peaks in the cultured cortical neurons overexpressed with GATA1. **(A)** Genes for unique peaks of H3K4me3 in the promoter regions were functionally categorized under the biological process. **(B)** Top 3,000 genes by peak score for unique peaks of H3K27me3 in the promoter regions were functionally categorized under the biological process. The horizontal axis shows the cluster number in enrichment score order. The vertical axis shows the negative of the base 10 logarithm of the Benjamini adjusted *p*-value. Node color represents the subcategory and node size represents the gene count. Benjamini <0.1 were considered significant. **(C)** Genes for unique peaks of H3K4me3 (upper) and H3K27me3 (lower) in the promoter regions were functionally categorized under KEGG pathway. The horizontal axis shows the enrichment score of each cluster. Node color represents the Benjamini adjusted p-value, while node size represents the gene count. Benjamini <0.1 were considered significant.

### 2.19 Sucrose preference test

The sucrose preference test was performed to observe anhedonic-like behavior. All mice were individually housed and exposed to a sucrose solution (1%, w/v) for adaptation for 24 h, followed by 24 h of water deprivation. Then, the mice were given two identical bottles containing 200 mL of sucrose solution (1%, w/v) and 200 mL of tap water for 16 h and given free access to two bottles. The sucrose and water intakes were measured by weighing all bottles before and after exposure to mice. The percentage of sucrose preference was calculated according to the following formula: consumed sucrose solution (g)/total consumed solution (sucrose + water) (g) × 100.

### 2.20 Forced swim test

A forced swim test was conducted for evaluating behavioral despair. Mice were placed individually into a cylinder (length 30 cm, diameter 10 cm) containing tap water up to a depth of 20 cm depth (24–25°C) and allowed to swim for 6 min. Total immobility times during the last 4 min of the testing period were recorded and analyzed manually. The mice were considered to be immobile when they floated in the water with no struggling and only demonstrated minor movement that was necessary to keep their heads above the water.

### 2.21 Confocal imaging and dendritic spine density measuring

Confocal images were acquired using confocal laser scanning microscopy (Nikon ECLIPSE Ti). Z-stack images were obtained with 60x/1.4 NA oil-immersion objectives. Microscope settings were as followed: 28.85 us pixel dwell, resolution 1,024 × 1,024 pixels, z-step 0.3 μm, unidirectional acquisition mode, and color depth 12 bit. Confocal images were initially deconvolved and analyzed using NIS-Elements software version 5.01. Dendritic spine density was calculated as total number of dendritic spines/dendrite length. Dendrite length was measured by using Image J. To get the pixel value for μm, the length of the scale bar on the image was measured using a line tool. After applying the pixel value for μm using a set scale tool, a line was drawn along the dendrite using the free-hand line tool. All dendritic spines were counted regardless of their morphology.

### 2.22 Statistical analysis

The data were shown as mean ± standard error of the mean (SEM) and analyzed using GraphPad Prism 7.0 (GraphPad Software Inc., San Diego, CA, United States). Comparative analyses were performed using unpaired t-tests and two-way Analysis of Variance (ANOVA) followed by Tukey’s multiple comparison test (*p* < 0.05). Before performing the comparative analysis, we identified outliers, which were defined as values that fall more than 1.5 times the interquartile range (IQR) above the third quartile or below the first quartile and the values detected as outliers were removed.

## 3 Results

### 3.1 Profiling of genes with histone modification in the promoter regions by GATA1 in the cultured cortical neurons

**Figure 2:**
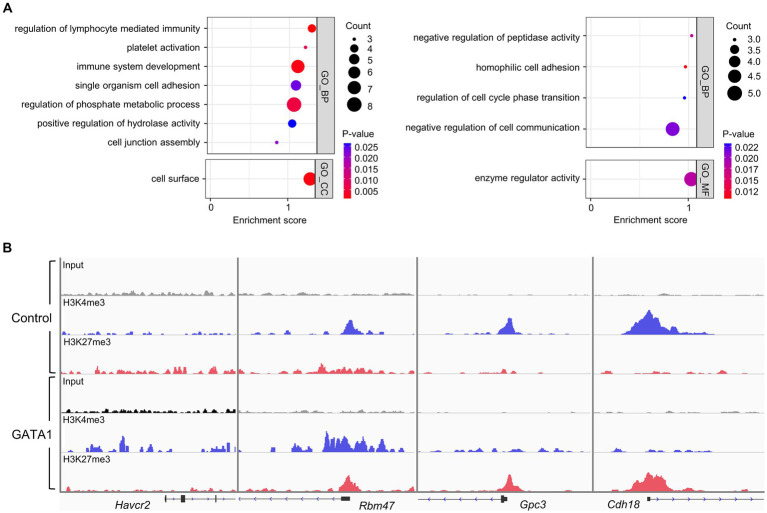
Functional annotation of DEGs in the cultured cortical neurons with overexpressed GATA1 and comparison with ChIPseq data. **(A)** Up-regulated genes (left panel) and down-regulated genes (right panel) were functionally categorized under biological process (GO_BP), cellular component (GO_CC), and molecular function (GO_MF). The horizontal axis shows the enrichment score of each cluster. Node color represents the p-value, and node size represents the gene count. *p*-values <0.05 were considered significant. **(B)** Representative peak images of differentially expressed genes (up-regulated genes; *Havcr2* and *Rbm47*, down-regulated genes; *Gpc3* and *Cdh18*) overlapped with ChIPseq result. IGV track displayed ChIPseq coverage for H3K4me3 (blue) and H3K27me3 (red) across the genome of each gene. Boxes represent exonic regions, and lines represent intronic regions. The arrows indicate the direction of transcription.

To explore the global alteration in gene expression by GATA1, we investigated the integrated gene expression profiles regulated by GATA1 in cortical neurons using high-throughput approaches (Supplementary Figure S1; Table S1). We performed ChIPseq for H3K4me3 and H3K27me3, which denote histone marks representing transcriptional activation and transcriptional repression, respectively. We identified 41,339 H3K4me3 peaks and 25,357 H3K27me3 peaks in cortical cultured neurons overexpressing GATA1 (Supplementary Table S2). We classified the genomic distribution of ChIPseq peaks into eight categories including promoter-TSS (transcription start site; from −1 kb to +100 bp), exon, intron, 5’UTR (5′-untranslated region), 3’UTR (3′-untranslated region), CpG-Island, TTS (transcription termination site; from −100 bp to +1 kb), and others (non-functional region such as simple repeat sequence; Supplementary Figure S2A). Among these, the peaks of the gene promoter region accounted for 5.7% (H3K4me3) and 20.5% (H3K27me3) in the GATA1-overexpressed cultured neurons. Then, we sorted out GATA1-specific unique peaks of H3K4me3 and H3K27me3 in the gene promoter regions (Supplementary Table S3). A GATA1-specific unique peak is a peak that is detected only in the GATA1-overexpressed group, i.e., a peak that is not in the control group. We identified 1,127 and 4,881 unique peaks, respectively, in the gene promoter region in cultured cortical neurons overexpressing GATA1 compared to the control (Supplementary Figure S2B). GO analysis showed the functional enrichment classification of unique peak genes, which might be transcriptionally regulated by GATA1 (Benjamini <0.1; Supplementary Table S4). To acquire the intuitive point of view, GO terms categorized in biological processes were divided into 10 subcategories, i.e., cell proliferation and differentiation, cellular process, developmental process, immune-related process, macromolecule processing, response to stimulus, signaling, synapse-related function, transport, and others. Among these subcategories, GATA1 might be involved in transcriptional activation of genes related to developmental process (39.58%), immune-related function (11.81%), and cell proliferation and differentiation (9.03%; Figure 1A; Supplementary Figure S3A). It could also act as a transcriptional repressor of genes related to macromolecule processing (31.25%), cellular process (25.00%), and developmental process (12.50%; Figure 2B; Supplementary Figure S3B). Interestingly, of the 10 categories, the GO terms classified as immune-related process were overrepresented only in the results of GO analysis of genes activated by GATA1, including angiogenesis (*Benjamini p* = 5.57 × 10^−10^), lymph vessel development (*Benjamini p* = 6.19 × 10^−5^), regulation of blood circulation (*Benjamini p* = 3.23 × 10^−4^), regulation of immune system process (*Benjamini p* = 2.48 × 10^−3^), cell adhesion (*Benjamini p* = 3.15 × 10^−8^), JAK–STAT cascade (*Benjamini p* = 1.22 × 10^−2^), and response to cytokine (*Benjamini p* = 4.36 × 10^−2^; Supplementary Table S4). Furthermore, KEGG pathway analysis showed that transcriptionally activated genes, which were GATA1-unique peak genes, were enriched in pathways associated with Maturity onset diabetes of the young (*Benjamini p* = 2.99 × 10^−4^), Leukocyte transendothelial migration (*Benjamini p* = 2.98 × 10^−2^), and ECM-receptor interaction (*Benjamini p* = 1.25 × 10^−2^; Figure 1C).

### 3.2 Differentially expressed mRNAs by GATA1 overexpression in the cultured cortical neurons

To profile the expression of transcriptome, we conducted mRNAseq analysis of GATA1-overexpressed cultured neurons. We identified that 113 mRNAs were differentially expressed in the cortical neurons overexpressing GATA1 compared to control (Supplementary Figure S4). These comprised 54 upregulated mRNAs and 59 downregulated mRNAs (Supplementary Table S5). GO analysis (*p*-value <0.05) indicated that upregulated genes were significantly overrepresented in the biological process (BP) associated with regulation of lymphocyte-mediated immunity (*p* = 9.87 × 10^−4^), platelet activation (*p* = 7.40 × 10^−3^), and immune system development (*p* = 1.95 × 10^−3^); and in the cellular component (CC) associated with cell surface (*p* = 1.73 × 10^−3^). Meanwhile, downregulated genes were significantly overrepresented in the BP associated with regulation of peptidase activity (*p* = 1.75 × 10^−2^), homophilic cell adhesion (*p* = 1.04 × 10^−2^) and regulation of cell cycle phase transition (*p* = 2.32 × 10^−2^); and in the molecular function (MF) associated with enzyme regulator activity (*p* = 1.81 × 10^−2^; Figure 2A; Supplementary Table S6). As in the ChIPseq analysis, subcategory classification showed that genes up-regulated by GATA1 overexpression may be involved in immune-related process and cellular process. We also confirmed that the ChIPseq peak showing transcriptional activation or repression in the promoter regions correlated with the expression pattern of several genes among differentially expressed genes (DEGs; Figure 2B). Hepatitis A virus cellular receptor 2 (*Havcr2*) was annotated to almost all immune-related GO terms. Havcr2, also known as T-cell immunoglobulin and mucin-domain containing-3 (Tim-3), is a cell surface receptor implicated in the immune responses to promote or inhibit (Leavy, 2008; Wolf et al., 2020). The RNA binding motif protein 47 (*Rbm47*) was annotated to immune system development (GO:0002520). Rbm47 as post-transcriptional regulator is involved in regulation of IL-10 expression in the B cell (Wei et al., 2019). Glypican 3 (*Gpc3*) and Cadherin18 (*Cdh18*) were annotated to regulation of peptidase activity (GO:0010466) and homophilic cell adhesion (GO:0007156), respectively. Gpc3 is a membrane protein involved in cell growth and division (Pan et al., 2013), but its function in the nervous system is unknown. Cdh18 is a cell adhesion protein involved in synaptic adhesion and has been implicated in several psychiatric diseases (Hawi et al., 2018).

**Figure 3:**
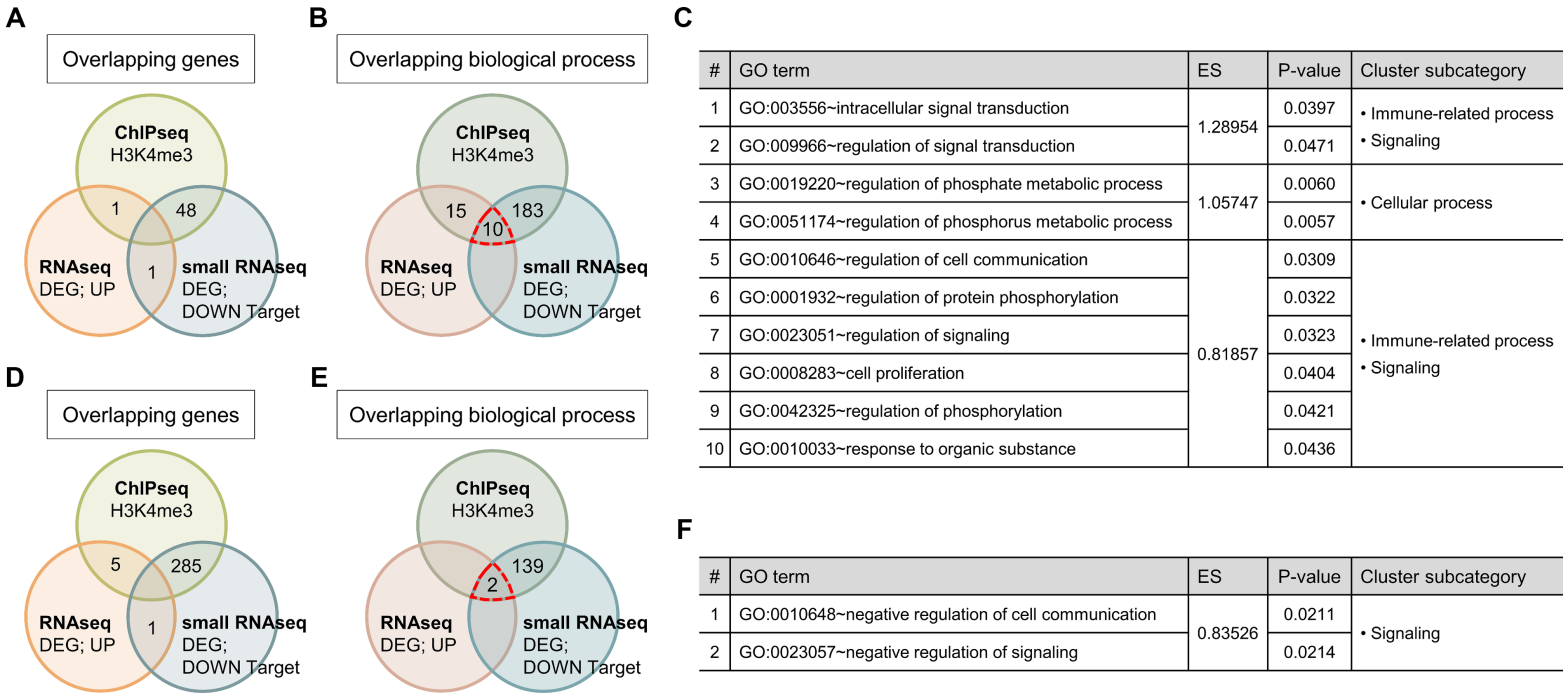
Relationships of the identified genes and GO terms between each omics data. **(A,D)** Venn diagram showing the number of overlapping genes that were activated or repressed by GATA1 overexpression between each sequencing data. **(B,E)** Venn diagram showing the number of overlapping GO terms in biological process between each sequencing data. ChIPseq, GATA1-unique peak genes; RNAseq, differentially expressed genes; small RNAseq, predicted target genes of differentially expressed miRNAs. **(C,F)** List of overrepresented GO terms overlapped in GO analysis of all sequencing data. GO terms were aligned in ascending order according to the enrichment score and p-value in the results of mRNAseq GO analysis.

### 3.3 Differentially expressed miRNAs by GATA1 overexpression in the cultured cortical neurons

Based on the small RNAseq analysis, we identified 82 miRNAs that were differentially expressed in the cortical neurons with overexpressed GATA1 compared to the control (Supplementary Figure S5A). These comprised 41 up-regulated miRNAs and 41 down-regulated miRNAs (Supplementary Table S7). To investigate the role of differentially expressed miRNAs (DEmiRNAs), we performed prediction analysis of miRNA targets using five miRNA databases including TargetScan, miRDB, miRanda, DIANA tools, and miRmap. The top 50 genes were obtained in each database, and the genes predicted in two or more databases were sorted (Supplementary Figure S5B; Table S8). Then, we performed GO analysis (Benjamini <0.1) and subcategory classification as described above. It was found that target genes of up-regulated miRNAs, which are expected to be decreased by GATA1 overexpression, were significantly overrepresented in BP associated with regulation of signaling (*Benjamini p* = 2.59 × 10^−8^), epithelium development (*Benjamini p* = 5.67 × 10^−7^), and regulation of macromolecule metabolic process (*Benjamini p* = 1.79 × 10^−8^; Supplementary Figure S6; Table S9). Subcategory classification indicated that target genes expected to be decreased were involved in development (34.78%), cellular process (13.04%), and metabolic process (11.59%; Supplementary Figure S7A). Furthermore, target genes of down-regulated miRNAs, which are expected to be increased by GATA1, were significantly overrepresented in BP associated with regulation of transcription (*Benjamini p* = 7.24 × 10^−8^), regulation of cellular component organization (*Benjamini p* = 2.98 × 10^−4^), and endodermal cell differentiation (*Benjamini p* = 3.00 × 10^−3^; Supplementary Figure S6; Table S9). Subcategory classification indicated that target genes expected to be increased were involved in developmental process (27.59%), macromolecule processing (13.79%), and signaling (12.07%; Supplementary Figure S7B). Of the 10 categories, the GO terms classified as immune-related process were overrepresented only in the results of GO analysis of genes activated by GATA1, among which is included angiogenesis (*Benjamini p* = 9.76 × 10^−3^), myeloid leukocyte differentiation (*Benjamini p* = 6.46 × 10^−2^), regulation of system process (*Benjamini p* = 9.40 × 10^−2^), and cell adhesion (*Benjamini p* = 4.00 × 10^−2^).

Finally, we conducted an integrative analysis of each omics data by comparing gene sets of GATA1-unique peak genes from the ChIPseq, DEGs from the RNAseq, and predicted target genes of DEmiRNAs from the small RNAseq. We also compared GO terms that were categorized in the biological processes from the GO analysis of the above gene sets. Although there were no overlapping genes across all sequencing data, several overlapping GO terms were overrepresented in the result of GO analysis (Figure 3). In the case of the biological process that might be activated by GATA1 overexpression, there were 10 common GO terms in all GO analysis results, and their subcategories were immune-related process, cellular process, and signaling (Figure 3C). Meanwhile, in the case of the biological process that might be repressed by GATA1 overexpression, there were two common GO terms that were subcategorized as signaling (Figure 3F). Comprehensively, the results of the sequencing data analysis indicated that GATA1 is extensively involved in various biological processes in the CNS. In particular, biological processes classified as immune-related processes were consistently overrepresented in the GO analysis of genes activated by GATA1. These results suggest that GATA1 might be involved in the transcriptional activation of genes associated with immune function.

**Figure 4:**
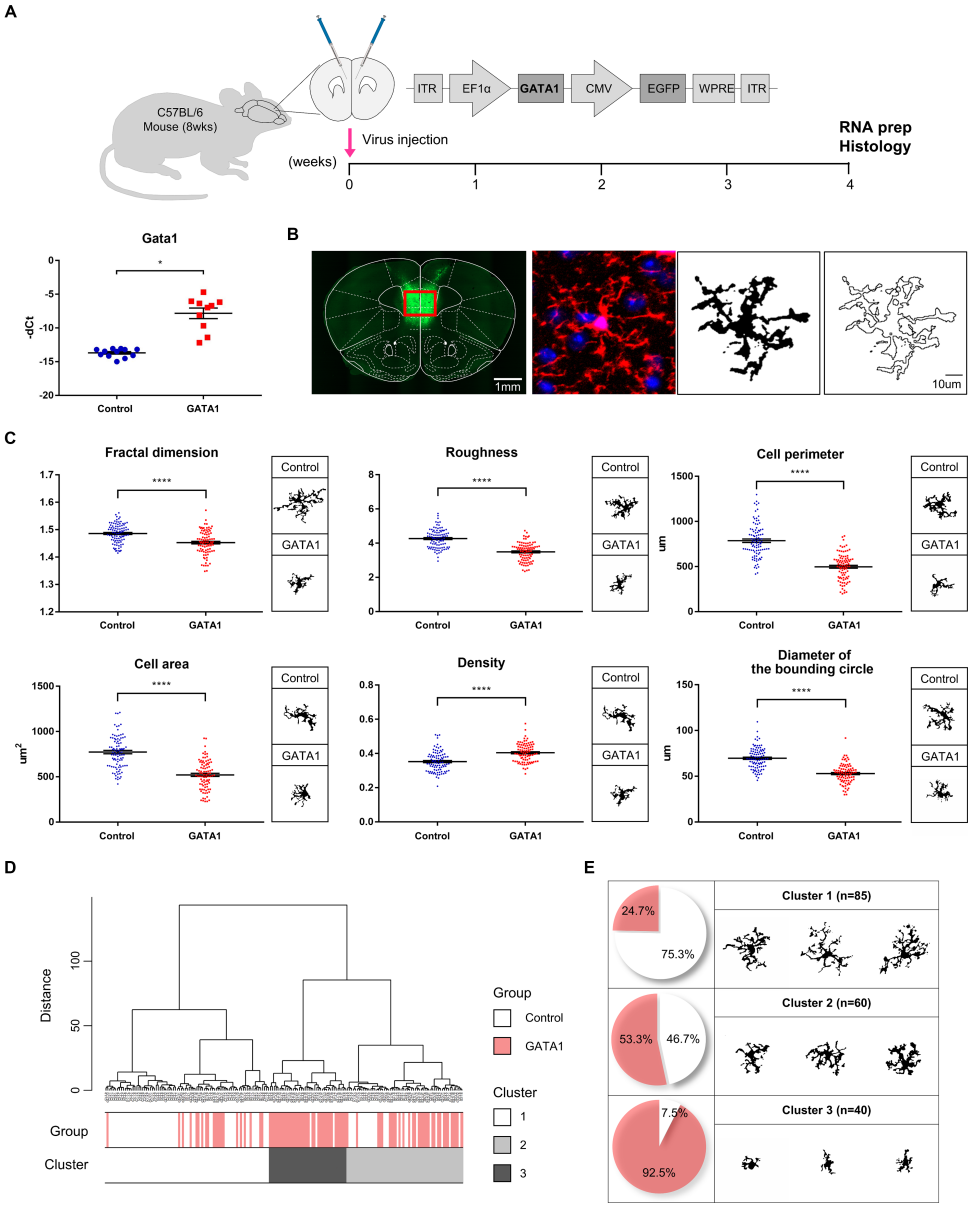
Microglial morphometric analysis. **(A)** Schematic illustration of the experimental timeline and confirmation of GATA1 overexpression by qRT-PCR. All Ct values were normalized to those of *Gapdh* (fold change = 58.405, *p* = 2.63 × 10^−5^). Data are shown as mean ± SEM (-dCt) of gene in control (*n* = 12) and GATA1 (*n* = 10) overexpressed mice. *p < 0.05 upon comparison of GATA1-overexpressed mice to the control using unpaired t-test. **(B)** Pre-processing of cell digital image. Fluorescent image transformed into a binary image comprising filled image and outlined image. **(C)** Comparison of morphological parameters between microglial cells in the Control and GATA1 group. Graph shows mean ± SEM of parameters of each cell on Control (*n* = 95) or GATA1 (*n* = 90). Asterisks indicate significant differences (*p* < 0.0001) by unpaired t-test. **(D)** Hierarchical cluster analysis of microglial cells. Dendrogram for 185 cells, where the abscissa represents individual cells, and the ordinate corresponds to the linkage distance measured by Euclidean distance. Clusters were color-coded white (cluster 1), light gray (cluster 2), and dark gray (cluster 3). **(E)** Number and percentage of cells belonging to each cluster and representative cell image of each cluster.

### 3.4 Morphometric analysis of microglia in the medial prefrontal cortex of GATA1 overexpression

As microglia are the primary immune cells in the CNS, we assessed microglia morphology to determine whether microglia are activated by GATA1. Through stereotaxic surgery, GATA1 was induced into the medial prefrontal cortex (mPFC) of mice brains. After 4 weeks of recovery, brain tissue was prepared and microglia were labeled using ionized calcium-binding adaptor protein-1 (Iba1), which are pan markers for all microglia (Figure 4A). For analysis, each cell was converted to a binary image comprising filled image and outlined image (Figure 4B). The morphological features of each cell were evaluated by measuring 15 parameters with the FracLac for Image J. These parameters can be divided into three groups depending on their character, where one represents complexity of the cell, another represents cell size, and the third represents circularity of the cell. As a result, for parameters belonging to the complexity group, fractal dimension (*p* = 5.25 × 10^−8^), roughness (*p* = 3.55 × 10^−18^), cell perimeter (*p* = 6.03 × 10^−24^), and convex hull perimeter (*p* = 1.03 × 10^−22^) were significantly decreased in the microglia in the mPFC of mice brains with overexpressed GATA1 compared to control (Figure 4C; Supplementary Figure S8). By contrast, there was no statistically significant difference in lacunarity between control and GATA1 (Supplementary Figure S8). For parameters belonging to the cell size group, cell area (*p* = 1.18 × 10^−18^), convex hull area (*p* = 1.28 × 10^−22^), diameter of the bounding circle (*p* = 1.86 × 10^−19^), maximum span across the convex hull (*p* = 2.78 × 10^−19^), and mean radius (*p* = 2.56 × 10^−21^) was significantly decreased while density (*p* = 3.28 × 10^−9^) was significantly increased in the microglia in the mPFC of mice brains with overexpressed GATA1 compared to control (Figure 4C; Supplementary Figure S8). This indicates that the microglia in the mPFC of mice brains with overexpressed GATA1 are more monotonous and smaller than that of the control, the process length is shorter, and the ramification is reduced. These morphological features are typically characteristic of activated microglia (Fernandez-Arjona et al., 2017). For parameters belonging to the circularity group, cell circularity (*p* = 5.14 × 10^−15^) was significantly increased in the microglia in the mPFC of mice brains with overexpressed GATA1 while others showed no significant difference between control and GATA1 (Supplementary Figure S8). If circularity has a value of 1, it is called a circular form (Walters et al., 2017). Although the microglia in both groups have an extremely low value of circularity, there are significant differences between control and GATA1, which indicate that microglia in the GATA1 group have an activated shape. Subsequently, we performed HCA with all parameter values and identified three clusters (Figure 4D). Cluster 1 contained more cells of the control group than those of the GATA1 group, whereas Cluster 3 showed the opposite trend (Figure 4E). A representative cell image of each cluster showed that the microglia contained in Cluster 3 have the form of activated microglia. Collectively, our data revealed that microglia were activated by GATA1 overexpression.

**Figure 5:**
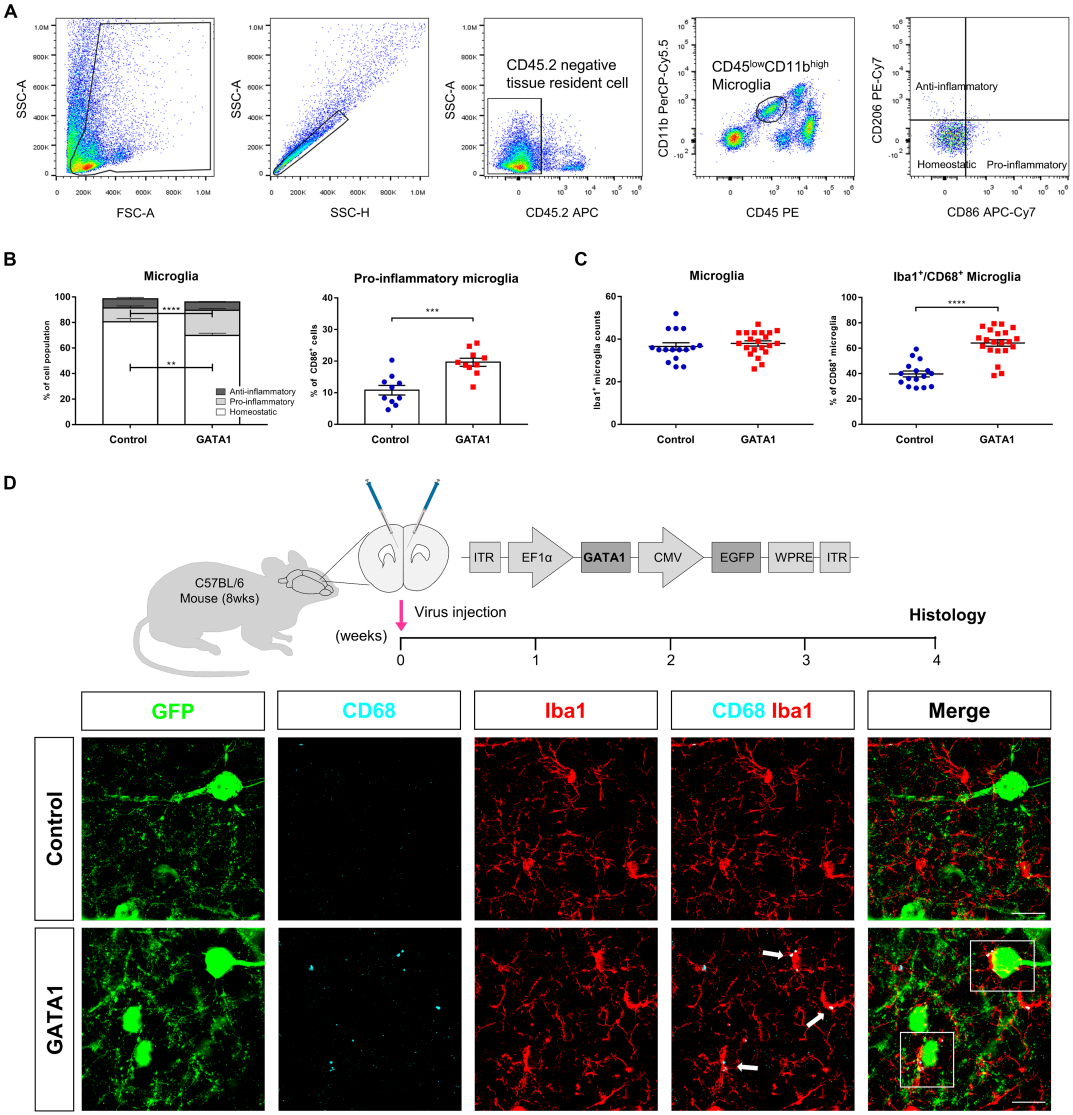
Phenotype of activated microglia in the brain of GATA1-overexpressed mice. **(A)** Gating strategy used for sorting of microglia and their subtype. **(B)** Graph displaying the calculated percentage of homeostatic (CD86^−^CD206^−^), pro-inflammatory (CD86^+^CD206^−^), and anti-inflammatory (CD86^−^CD206^+^) microglia. Pro-inflammatory microglia were significantly increased in the brain of GATA1-overexpressed mice, while homeostatic microglia were significantly decreased. Data shown as mean ± SEM (percentage) in control (*n* = 10) and GATA1 (*n* = 10). **(C)** Quantitative analysis of Iba1^+^CD68^+^ co-localized cells. CD68^+^ microglia were increased in the brain of GATA1-overexpressed mice, while there is no difference in total number of microglia. Data shown as mean ± SEM (percentage) in control (*n* = 16) and GATA1 (*n* = 21). **(D)** Immunofluorescence detection of microglial cells in the mPFC infected with Control or GATA1 virus (scale bar = 20 μm). Microglial cells were labeled with Iba1 (red) and CD68 (cyan). GFP signal represents virus-infected cells. Merge images represent the interaction between activated microglia and neurons (white box). *p < 0.05 upon comparison of GATA1-overexpressed mice to the control using unpaired t-test.

### 3.5 Characterization of activated microglia by GATA1 overexpression

Microglia phenotypes are divided into pro-inflammatory and anti-inflammatory microglia depending on their characteristics including surface marker, secretory molecules, and their function (Zhang et al., 2018). To examine the phenotype of activated microglia by GATA1 overexpression, we performed flow cytometry analysis. We labeled the cell with specific surface markers and defined each population by following a gating strategy (Figure 5A). At first, “gate” was defined as whole cells except debris and then discriminated single cells from doublets. Subsequently, we gated CD45.2^−^ cells, which are pan markers for hematopoietic cells, to discriminate brain resident cells. Microglia were identified to exhibit lower expression of CD45 and higher expression of CD11b. Finally, we used CD86, a pro-inflammatory microglia marker because it was a co-stimulatory receptor that generates signals after MHC II activation (Jurga et al., 2020), and CD206, an anti-inflammatory microglia marker, to analyze the subpopulation of microglia. It was found that pro-inflammatory microglia, presented as CD86^+^CD206^−^ cells, were significantly increased (*p* = 3.12 × 10^−4^) in the brain of GATA1-overexpressed mice while there were no changes in anti-inflammatory microglia, presented as CD86^−^CD206^+^ cells (Figure 5B). These results indicated that microglia were activated toward the pro-inflammatory phenotype by GATA1 overexpression. Consequently, we assessed the CD68^+^ microglia cells through fluorescent immunohistochemistry. CD68, a common marker for macrophage lineage cells, labels the lysosome and is therefore commonly considered a marker of activated phagocytic microglia (Hopperton et al., 2018). Accordingly, we quantified Iba1^+^ CD68^+^ co-localized cells in the brain with overexpressed GATA1 compared to the control. Immunohistochemical analysis revealed that the total number of microglia, marked with Iba1, is similar between both groups, whereas Iba1^+^ CD68^+^ microglia were significantly increased (*p* = 6.10 × 10^−8^) in the brain of GATA1-overexpressed mice (Figure 5C). Furthermore, a high magnification image showed that these microglia activated by GATA1 interact with surrounding neurons (Figure 5D).

**Figure 6:**
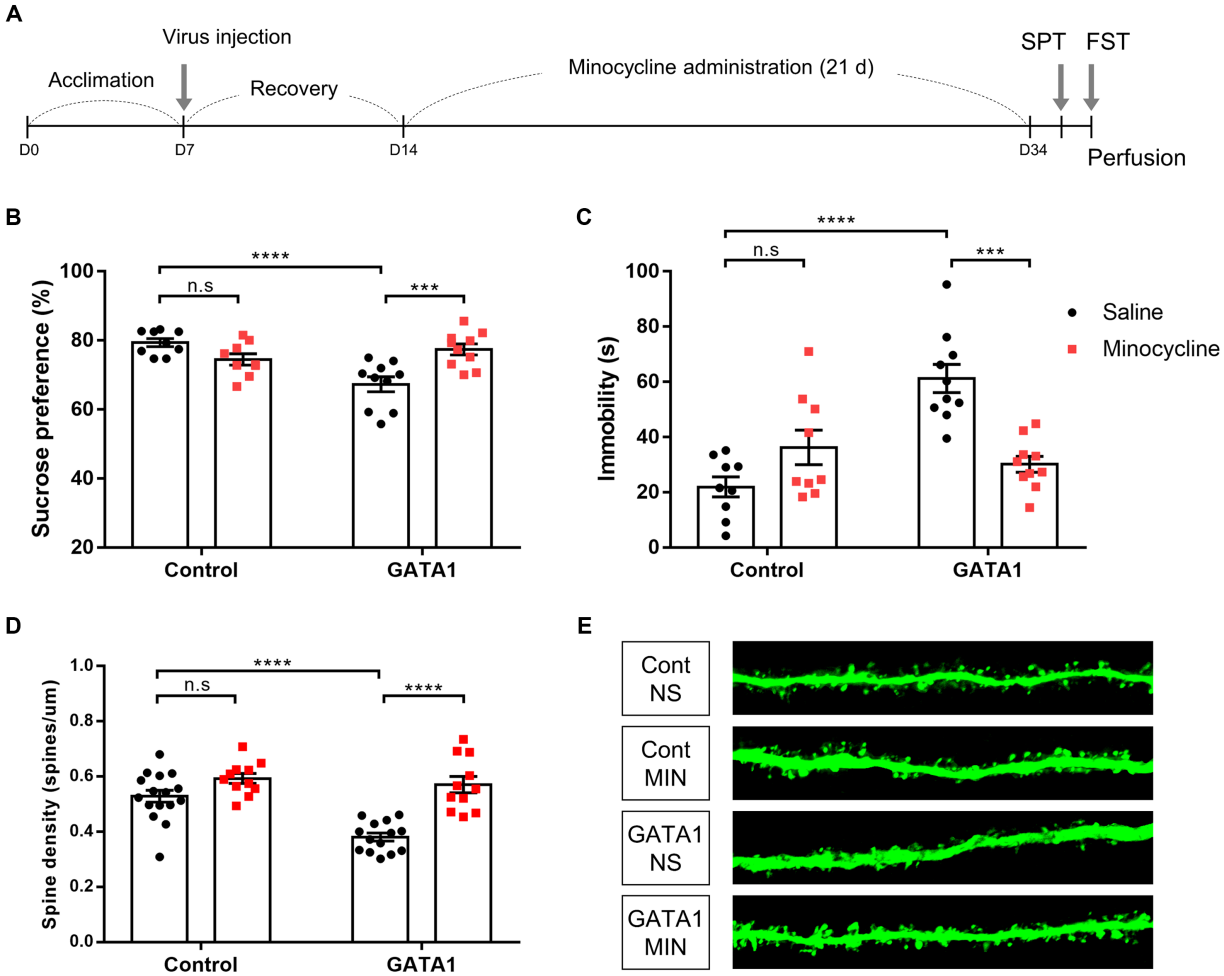
Effects of minocycline treatment on behavior and dendritic spine analysis in GATA1-overexpressed mice. **(A)** Experimental schedule of the study design. After acclimation for a week, mice were subjected to stereotaxic surgery and recovery over a week. After minocycline administration for 3 weeks, behavior tests were performed. **(B)** Sucrose preference, which represents anhedonic behavior, was measured in the SPT. **(C)** Immobility times, which represent behavioral despair, were measured in the FST. Data shown as mean ± SEM (sucrose preference or immobility time) in CT_NS (*n* = 9), CT_MIN (*n* = 9), GT_NS (*n* = 10), and GT_MIN (*n* = 10). (D, E) Effect of spine density on minocycline treatment in the mPFC of GATA1-overexpressed mice (D), and representative confocal image (E). Data shown as mean ± SEM (spines/µm) in CT_NS (*n* = 16), CT_MIN (*n* = 11), GT_NS (*n* = 14) and GT_MIN (*n* = 11). Comparative analyses were performed using two-way ANOVA followed by Tukey’s multiple comparison test (*p < 0.05, **p < 0.01, ***p < 0.0001, ****p < 0.0001).

To better understand the inflammatory state associated with GATA1, we examined the expression of cytokine genes using qPCR. Results revealed that the expression of interleukin-1 beta (*IL-1β*; fold change = 1.518, *p* = 0.0137) and tumor necrosis factor alpha (*TNFα*; fold change = 1.368, *p* = 0.0183) were significantly upregulated and there was a tendency of increased expression of interleukin-1 alpha (*IL-1α*; fold change = 1.293, *p* = 0.0535), interferon gamma (*IFNγ*; fold change = 1.280, *p* = 0.3136), and transforming growth factor beta (*TGFβ*; fold change = 1.280, *p* = 0.0654) in the mPFC of GATA1-overexpressed mice (Supplementary Figure S9). Collectively, these results suggest that GATA1 overexpression induced inflammation by activating microglia.

### 3.6 Inhibition of microglial activation using minocycline administration blocks the depressive-like behavior induced by GATA1 overexpression

We hypothesized that the influence of GATA1 on synapse loss and depressive-like behavior would be blocked by inhibiting the activation of microglia. To examine this, mice were divided into four groups. We used minocycline, a drug that has been commonly used as an inhibitor of microglial activation and has BBB-penetrating and immune-suppressing properties (Kobayashi et al., 2013). All mice were subjected to stereotaxic surgery to infuse either the GATA1 or control virus. After recovery over 1 week, all mice received the same dose of minocycline or normal saline once daily for 21 days (Figure 6A). SPT and FST were used for measuring anhedonic behavior and despair, respectively. First, we confirmed that the effect of GATA1 overexpression was reproduced in mice, as shown in a previous study that demonstrated that depressive-like behavior was induced by GATA1 in rats (Kang et al., 2012). GATA1-overexpressed mice injected with normal saline (Group 3; GT_NS) showed significantly decreased sucrose preference (two-way ANOVA, F_1,34_ = 7.163, *p* = 0.0114 (gene), F_1,34_ = 19.3, *p* = 0.0001 (interaction between gene and drug), Tukey’s multiple comparison, CT_NS and GT_NS; *p* < 0.0001, CT_MIN and GT_NS; *p* = 0.0258) and increased total immobility time (two-way ANOVA, F_1,34_ = 12.92, *p* = 0.001 (gene), F_1,34_ = 24.19, *p* < 0.0001 (interaction between gene and drug), Tukey’s multiple comparison, CT_NS and GT_NS; *p* < 0.0001, CT_MIN and GT_NS; *p* = 0.0029; Figures 6B,C). Compared to this group, GATA1-overexpressed mice injected with minocycline showed no depressive-like behavior, as they had high sucrose preference (Tukey’s multiple comparison, GT_NS and GT_MIN; *p* = 0.0007) and lower total immobility time (Tukey’s multiple comparison, GT_NS and GT_MIN; *p* = 0.0001; Figures 6B,C). Subsequently, we performed the spine density analyses. We found that spine density was significantly decreased in the mPFC of GATA1-overexpressed mice injected with normal saline (two-way ANOVA, F_1,48_ = 15.53, *p* = 0.0003 (gene), F_1,48_ = 34.85, *p* < 0.0001 (drug), F_1,48_ = 8.491, *p* = 0.0054 (interaction between gene and drug), Tukey’s multiple comparison, CT_NS and GT_NS; *p* < 0.0001, CT_MIN and GT_NS; *p* < 0.0001; Figures 6D,E). By contrast, there is no reduction of spine density in the mPFC of GATA1-overexpressed mice injected with minocycline (Tukey’s multiple comparison, GT_NS and GT_MIN; *p* < 0.0001; Figures 6D,E). These results indicated that depressive-like behavior and synapse loss by GATA1 overexpression could be attributed to activated microglia.

## 4 Discussion

In the present study, we examined the global alteration of gene expression affected by GATA1 overexpression in mouse cortical neurons. Through the integrative analysis of ChIPseq, mRNAseq, and small RNAseq, we have identified that GATA1 is involved in a wide variety of biological processes and specifically in the transcriptional activation of genes related to immune response. Using the results of the multi-omics data analysis, we focused on immune activation by the increased level of GATA1 in the CNS, which was observed in the dlPFC of patients with depression (Kang et al., 2012). First, we considered the biological processes that were consistently overrepresented in the GO analysis in each of the multi-omics data. Subcategory classification was used for a more intuitive organization of GO terms depending on their functional characteristics. We identified that GO terms classified as immune-related functions appeared in the GO analysis of genes activated by GATA1 (Supplementary Figures S3, S7). In addition, GO analysis of mRNAseq revealed that genes up-regulated by GATA1 overexpression were significantly enriched in biological processes associated with immune-related functions. Second, we examined the biological processes that overlapped in the GO analysis of all sequencing data. Results showed that overlapping biological processes across GO analysis of genes that might be activated by GATA1 were associated with immune-related processes (Figure 3). These results suggested that GATA1 might be involved in the transcriptional activation of immune-related genes in the CNS. However, we found that there were few genes that overlapped in common when comparing the set of genes whose transcription may be regulated by GATA1 in each of the sequencing data. This phenomenon was more so in the relationship between mRNAseq and other sequencing data. Given that chromatin modification and miRNAs were associated with epigenetic regulation of gene expression in common, there may be more overlapped genes between them. Furthermore, recent studies have revealed that there was a temporal desynchronization between changes in chromatin state and gene expression (Ma et al., 2020; Kiani et al., 2022). Considering the above, we speculated that it was difficult to identify genes showing common expression patterns in the multi-omics data obtained from the same timeline.

For a long time, stress has been recognized as a factor affecting human health and disease, and it has been implicated in the development of several diseases including cancer, inflammatory disease, and mental disorder (Slavich, 2016). Specifically, stress can cause structural and functional changes in the CNS, which are highly related to the onset of psychiatric diseases including depression, post-traumatic stress disorder, and anxiety (Yaribeygi et al., 2017). In the previous study, it was demonstrated that GATA1 functions as a transcription repressor of synapse-related genes, which is a cause of synapse loss (Kang et al., 2012). Cultured cortical neurons infected with GATA1 virus decreased the complexity of the dendritic arbor and the number of spines compared to the control virus (Kang et al., 2012). We demonstrated that synaptic atrophy induced by GATA1 overexpression observed in cultured cortical neurons also appears reproducibly in the brain of GATA1-overexpressed mice. It was revealed that spine density was significantly decreased in the mPFC of GATA1-overexpressed mice brains. Furthermore, GATA1 was demonstrated to be a stress-inducible gene in the brain and could also induce depressive-like behavior (Kang et al., 2012). Stress responses occur through the interconnection of multiple systems including the endocrine, immune, and nervous systems (Menard et al., 2017). The hypothalamic–pituitary–adrenal (HPA) axis is one of the core systems of the stress response in mammals (Smith and Vale, 2006). The final product of this axis is a glucocorticoid (cortisol in humans; corticosterone in rodents), which plays a role in inducing several physiological changes for the bodily defense to stress (Everly and Lating, 2013). Although glucocorticoid originally has an anti-inflammatory property, prolonged stress leads to glucocorticoid imbalance caused by hyperactivation of the HPA axis, which further leads to activation of the immune system toward a pro-inflammatory effect (Godoy et al., 2018; Takahashi et al., 2018). Dysregulation of the HPA axis has been implicated in the pathophysiology of depression, and neuroinflammation has been considered among the mechanisms that exacerbate depression (Iob et al., 2020). During the inflammatory response, innate immune cells produce various inflammatory mediators including cytokines and chemokines (Felger and Lotrich, 2013). Cytokines are an element of neuroimmune communication as well as important mediators of immune response (Trakhtenberg and Goldberg, 2011). The meta-analyses revealed that peripheral levels of inflammatory cytokines were altered in patients with depression compared to healthy control (Dowlati et al., 2010; Kohler et al., 2017; D'Acunto et al., 2019). In addition, sickness behavior triggered by pro-inflammatory cytokines produced by activated peripheral immune cells shared several symptoms with depression (Dantzer, 2009). These clinical data support the notion that inflammatory cytokines play an important role in the onset and maintenance of depression as mediators that impact neurotransmitter signaling, neuroendocrine signaling (e.g., HPA axis), and the neurotrophic system (Himmerich et al., 2019; Woelfer et al., 2019).

As the brain’s innate immune cells in the CNS, microglia are a source of cytokines and have an important role in health as well as under disease conditions (Li and Barres, 2018). Under normal physiological conditions, microglia are generally in the resting or inactive state and perform homeostatic functions from regulation of neuronal survival to maintaining synaptic networks (Yin et al., 2017). They can actively move and reshape their processes by extending and retracting, which helps in monitoring and surveying their surrounding environment (Shastri et al., 2013). However, when pathologic insult occurs and homeostasis is disturbed in the brain (e.g., under chronic stress conditions), microglia are activated accompanied by phenotype change (Boche et al., 2013). The features of activated microglia include morphological alteration, characterized by large cell bodies and fewer processes, and functional changes, which are expressions of many cytokines and receptors (Boche et al., 2013). A growing body of research suggests that microglial activation is associated with the etiology of depression, which is supported by clinical and preclinical studies (Jia et al., 2021). Post-mortem study and positron emission tomography (PET) imaging for depression have found that changes in microglia have been detected in the brain regions involved in depression, and these changes are correlated with the severity of depressive episodes (Steiner et al., 2011; Torres-Platas et al., 2014; Setiawan et al., 2015; Li et al., 2018). Furthermore, preclinical studies have provided evidence supporting the correlation between the activation of microglia and depression. In various animal models of depression, activation of microglia is observed in several brain regions, concomitant with high levels of pro-inflammatory cytokines (Tynan et al., 2010; Liu et al., 2019). Therefore, these activated microglia have been considered key immune effector cells in the pathophysiology of depression (Singhal and Baune, 2017). Our results showed that microglia were activated into phenotype with pro-inflammatory properties by GATA1 overexpression. These activated microglia would be key factors in the inflammatory environment. Furthermore, the expression of inflammatory cytokine genes was altered in the brains of GATA1-overexpressed mice. The mRNA expression of IL-1β and TNFα was significantly increased in the mPFC of GATA1-overexpressed mice. Several clinical studies showed that the blood levels of these cytokines have been increased in patients with depression (Thomas et al., 2005; Osimo et al., 2020; Das et al., 2021). Increased levels of IL-1β influenced HPA axis hyperactivation, decreased hippocampal neurogenesis, and synaptic dysfunction (Goshen and Yirmiya, 2009; Liu and Quan, 2018; Nemeth and Quan, 2021). TNFα could also cause synapse loss following cytotoxic damage, as well as neuronal death, which is mediated by microglial phagocytosis (Neniskyte et al., 2014; Olmos and Llado, 2014). The expression of TGFβ was also increased in brains with overexpressed GATA1. As an anti-inflammatory cytokine, this cytokine has been known to display neuroprotective effects (Dobolyi et al., 2012; Lobo-Silva et al., 2016). We speculate that an increase in anti-inflammatory cytokines may have resulted from an immunomodulatory response to neuroinflammation. Although not in the CNS, there were several reports that GATA1 was involved in the expression of inflammatory mediators in the peripheral system (Masuda et al., 2004; Nigo et al., 2006; El Gazzar, 2007; Cole et al., 2010; Liu et al., 2018; Abdul Qayum et al., 2019). These findings might support the notion that GATA1 is involved in the inflammatory process.

In conclusion, our study revealed that GATA1 could drive an inflammatory state through the activation of microglia toward the pro-inflammatory phenotype, and neuroinflammation by GATA1 might cause synapse loss and depressive-like behavior. We could only demonstrate the phenomena of microglia changes that occur when GATA1 is overexpressed. Further studies will be required to determine the mechanisms by which GATA1 activates microglia. In this regard, we hypothesize that changes in gene expression induced by GATA1 overexpression in cultured cortical neurons might activate microglia. In particular, genes included in biological processes classified as immune-related processes that are overrepresented in the GO analysis of genes activated by GATA1 could be candidates. These include antigen presentation molecules such as major histocompatibility complex (MHC) class I and complement components. In developing brains, it was well known that microglia interact with neuronal MHC I, which leads to synapse elimination for functional consequences (Miyamoto et al., 2013; Cserép and Pósfai, 2021). In addition, neuronal expression of MHC I has been implicated in CNS diseases by playing a neuroinflammatory role (Cebrián et al., 2014). Through the previous studies and our multi-omics data, we speculate that GATA1 could induce increasing expression of MHC I in synaptic dendrites. This MHC I might interact with surrounding surveillant microglia. Such microglia-synapse interactions could lead to microglial activation that acquires phagocytic activity and release of pro-inflammatory mediators. Further studies would be needed to explain this. Another challenge is to explore whether other peripheral immune cells, such as T cells, are involved in neuroinflammation-induced synaptic atrophy or depressive-like behavior.

## Data availability statement

The datasets presented in this study can be found in online repositories. Raw data can be accessible at the Gene Expression Omnibus (GEO) repository under accession number (GSE 181679).

## Ethics statement

The animal study was approved by the Committee on Institutional Animal Care and Use of Chung-Ang University. The study was conducted in accordance with the local legislation and institutional requirements.

## Author contributions

KC: Writing – original draft. JL: Writing – original draft. GK: Writing – original draft. YL: Writing – original draft. HK: Funding acquisition, Writing – review & editing, Conceptualization.

## Footnotes

^1^ http://homer.ucsd.edu/homer/ngs/peaks.html

^2^ http://homer.ucsd.edu/homer/ngs/annotation.html

^3^ http://www.bioinformatics.babraham.ac./uk/projects/fastqc/

^4^ http://bowtiebio.sourceforge.net/

^5^ http://www.mirdb.org/

^6^ http://www.microrna.org

^7^ http://diana.imis.athena-innovation.gr/DianaTools/

^8^ http://mirmap.ezlab.org/app/

^9^ http://www.targetscan.org/

^10^ https://davidd.ncifcrf.gov/

^11^ http://bsi.kist.re.kr

^12^ https://imagej.net/Fiji
