# Supplementary material for: Recovery of synaptic loss and depressive-like behavior induced by GATA1 through blocking of the neuroinflammatory response
Source: Front Cell Neurosci. 2024 May 9;18:1369951. doi: 10.3389/fncel.2024.1369951 (PMC11112091; doi:10.3389/fncel.2024.1369951)
Supplement: Supplementary file 1 [file Data_Sheet_1.docx]

**Supplementary Information**

**Recovery of synaptic loss and depressive-like behavior induced by GATA1 through blocking of the neuroinflammatory response**

Koeul Choi, Joonhee Lee, Gukdo Kim, Younghyun Lim, and Hyo Jung Kang*

Department of Life Science, Chung-Ang University, Seoul, Korea

* Hyo Jung Kang

Email: hyokang@cau.ac.kr

**Supplementary Figures**

**
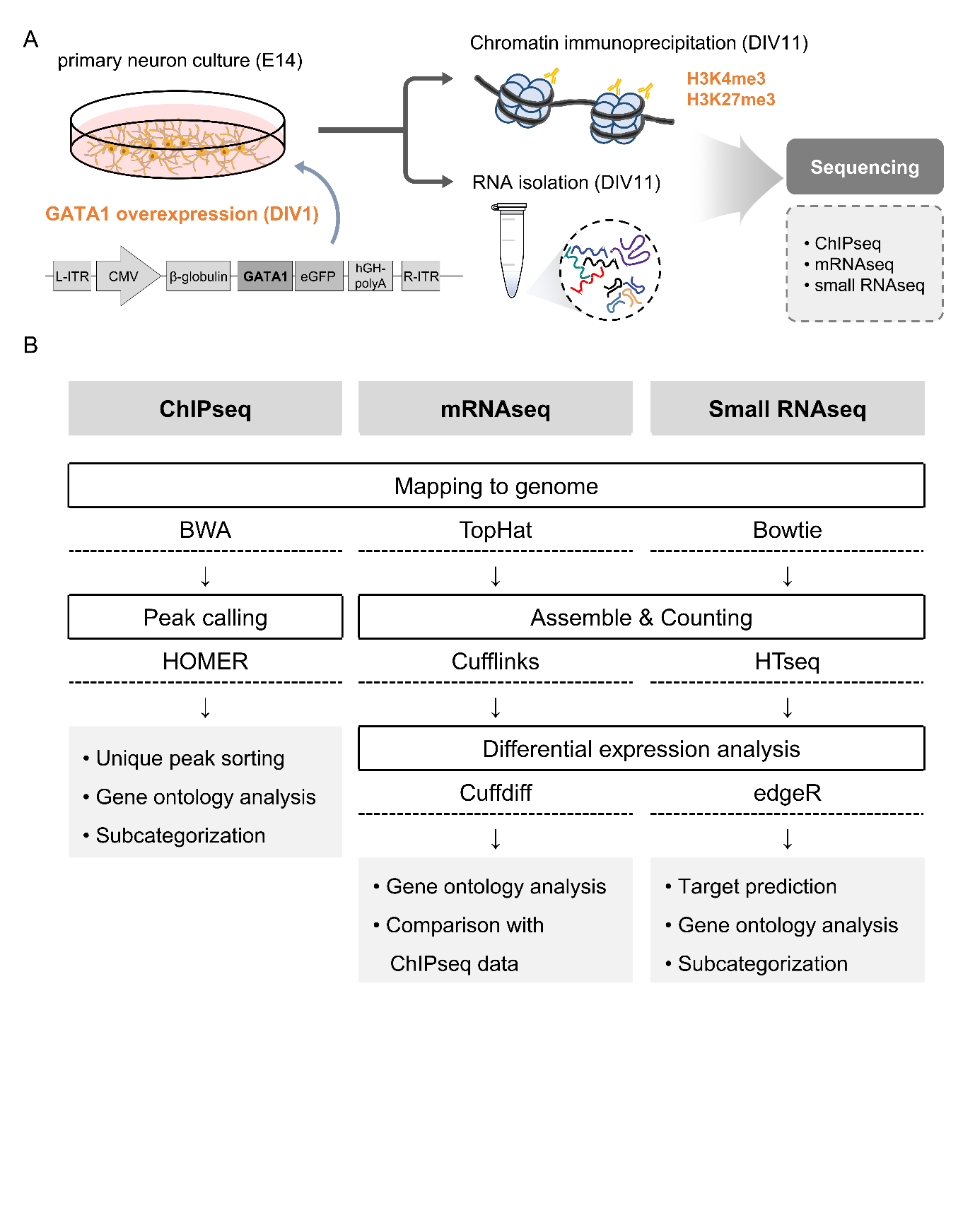
**

**Supplementary Figure S1.** Overview of experimental design and analysis workflow. **(A)** Experimental overview. **(B)** Workflow for the analysis of sequencing data.

**
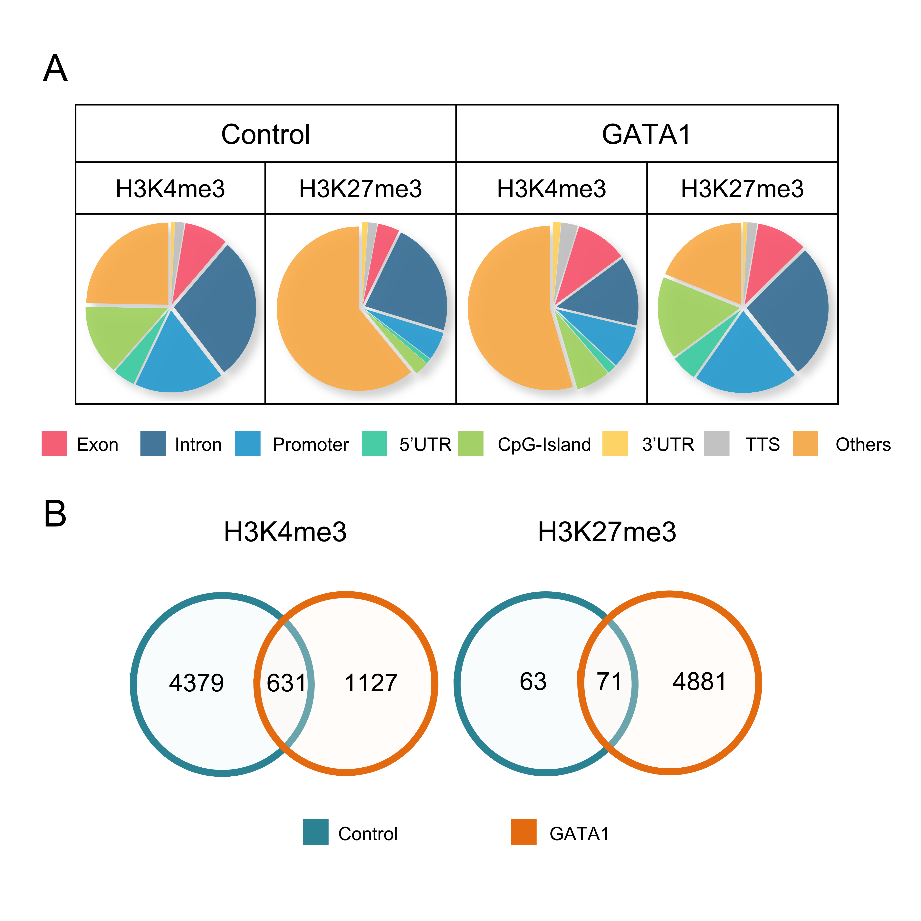
**

**Supplementary Figure S2.** Identification of unique peaks in the promoter regions of GATA1 overexpressed cultured cortical neurons. **(A)** Pie chart indicating the genomic distribution of ChIPseq peaks relative to gene location. **(B)** Venn diagram of H3K4me3 (left) and H3K27me3 (right) peaks comparison between control and GATA1 in the promoter regions. In the H3K4me3 peaks, a total of 1127 peaks are GATA1-unique. In the H3K27me3 peaks, a total of 4881 peaks are GATA1-unique.


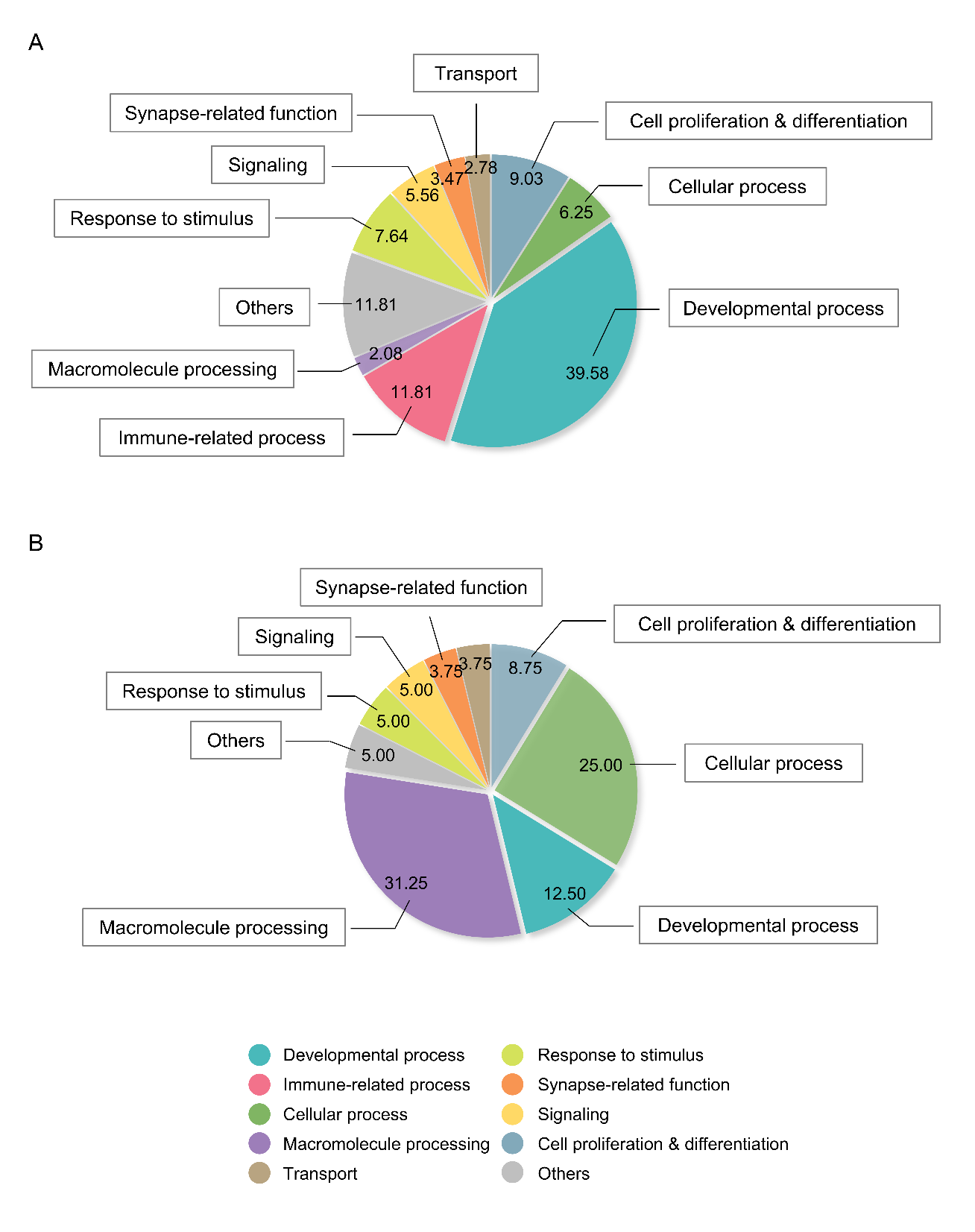


**Supplementary Figure S3.** Subcategorization of Gene Ontology (GO) terms in the GO analysis of GATA1 unique peak genes in the promoter regions. Pie chart showing the subcategorization of GO terms overrepresented in GO analysis of genes for GATA1 unique peaks of H3K4me3 **(A)** and H3K27me3 **(B)**. Numbers on pie chart represent the percentage of each category.


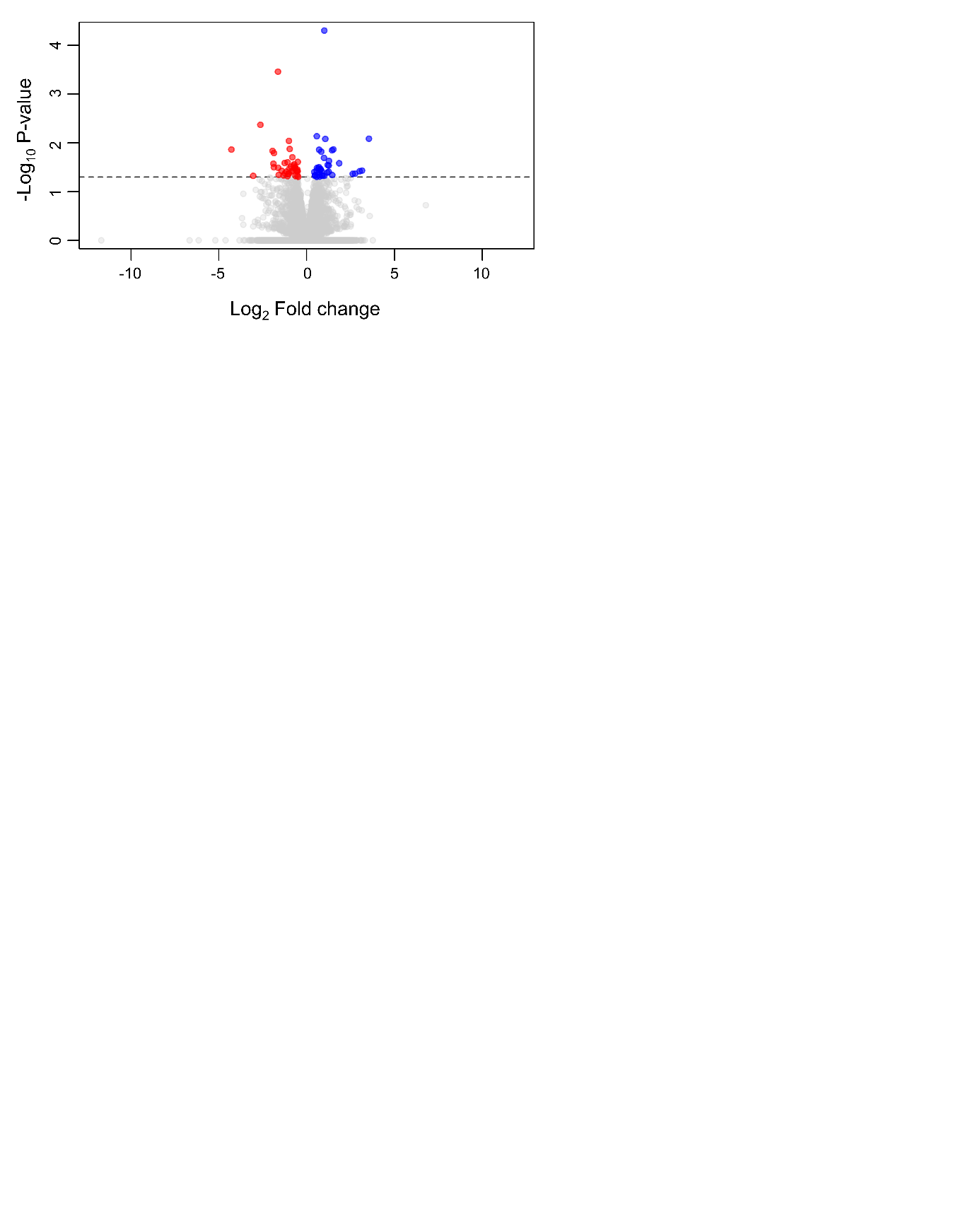


**Supplementary Figure S4.** Identification of differentially expressed genes (DEGs) in mRNAseq. Volcano plot of DEGs in the cultured cortical neurons overexpressed with GATA1. Genes with infinite fold change were excluded. The horizontal axis shows the base 2 logarithm of the fold change. The vertical axis shows the negative of the base 10 logarithm of the P-value. Significantly up-regulated genes are shown in blue, while significantly down-regulated genes are shown in red. P-values < 0.05 were considered to be differentially expressed genes.

**
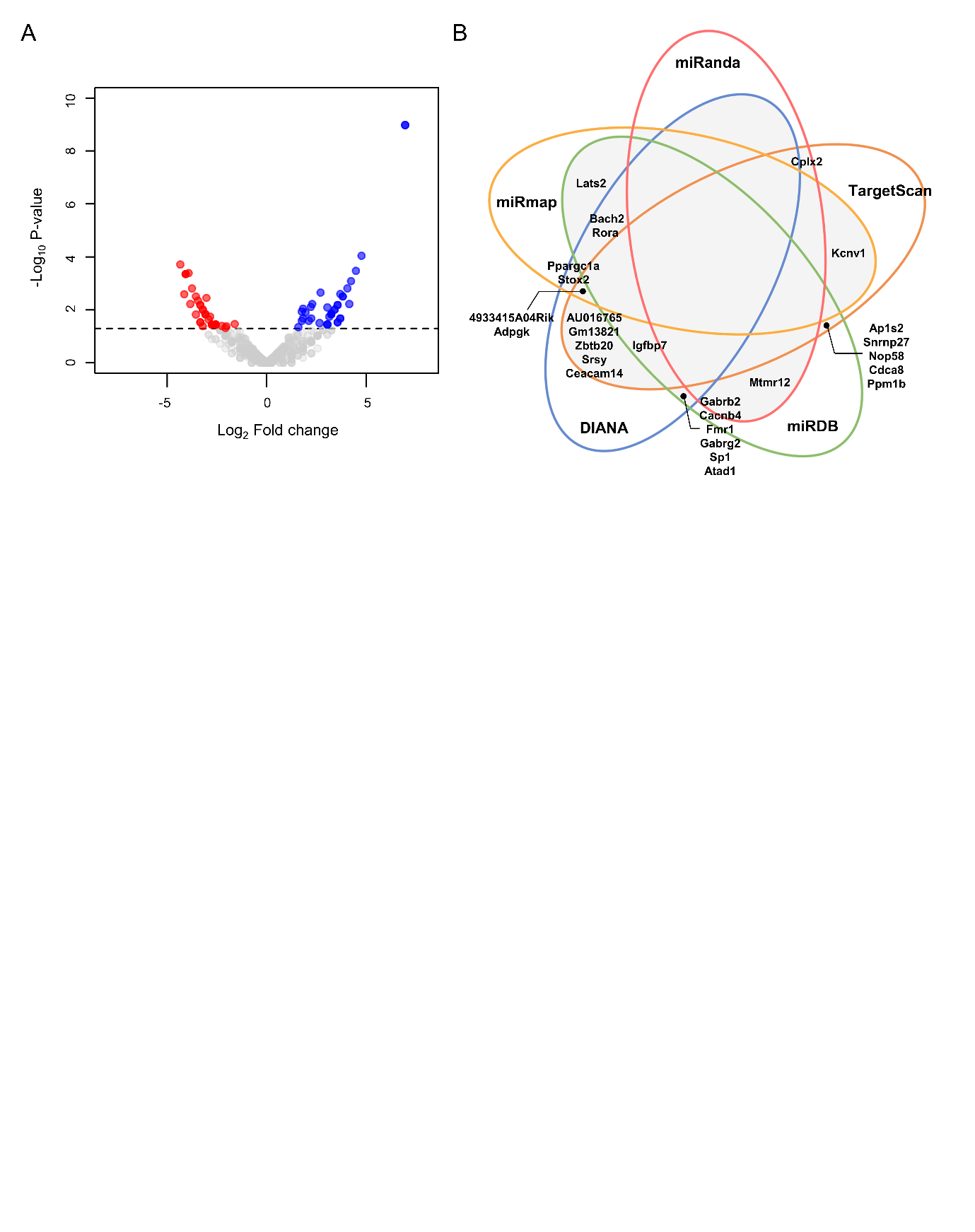
**

**Supplementary Figure S5.** Identification of differentially expressed miRNAs (DEmiRNAs) in the small RNAseq and their target prediction analysis. **(A)** Volcano plot of DEmiRNAs in the cultured cortical neurons in which GATA1 is overexpressed. The horizontal axis shows the base 2 logarithm of the fold change. The vertical axis shows the negative of the base 10 logarithm of the P-value. Significantly up-regulated miRNAs are shown in blue, while significantly down-regulated miRNAs are shown in red. P-values < 0.05 were considered to be differentially expressed miRNAs. **(B)** Venn diagram of the predicted target genes of DEmiRNA (mmu-mmiR-466b-5p; miRNA ranked first in DEmiRNAs) obtained from five online databases. The gray portion includes genes that were obtained from two or more databases.

**
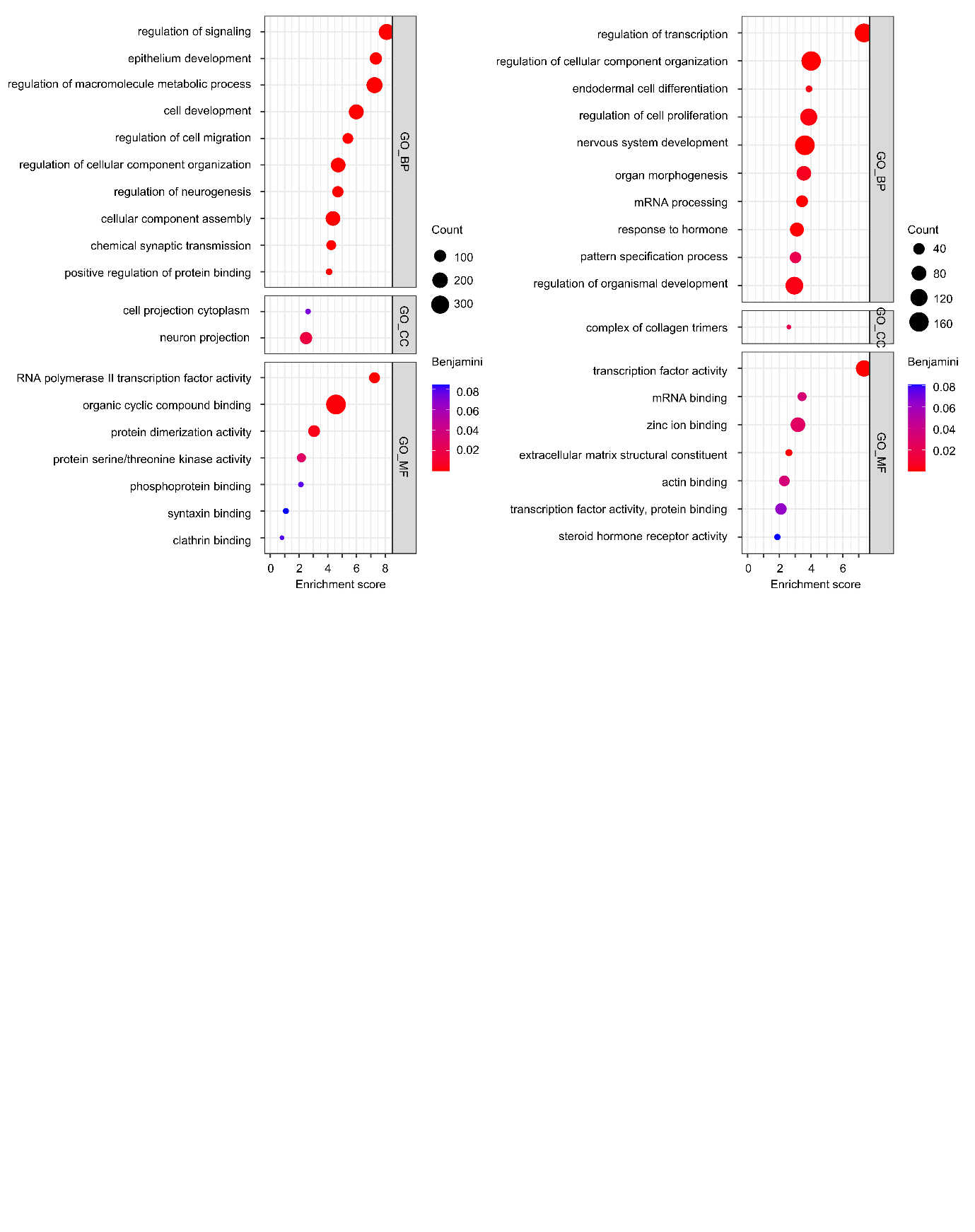
**

**Supplementary Figure S6.** Functional annotation of predicted target genes of DEmiRNAs in the cultured cortical neurons in which GATA1 is overexpressed. Target genes of up-regulated miRNAs (left panel) and down-regulated miRNAs (right panel) were functionally categorized under biological process (GO_BP), cellular component (GO_CC), and molecular function (GO_MF). The horizontal axis shows the enrichment score of each cluster. Node color represents the Benjamini adjusted P-value, and node size represents the gene count. Benjamini < 0.1 were considered significant.

**
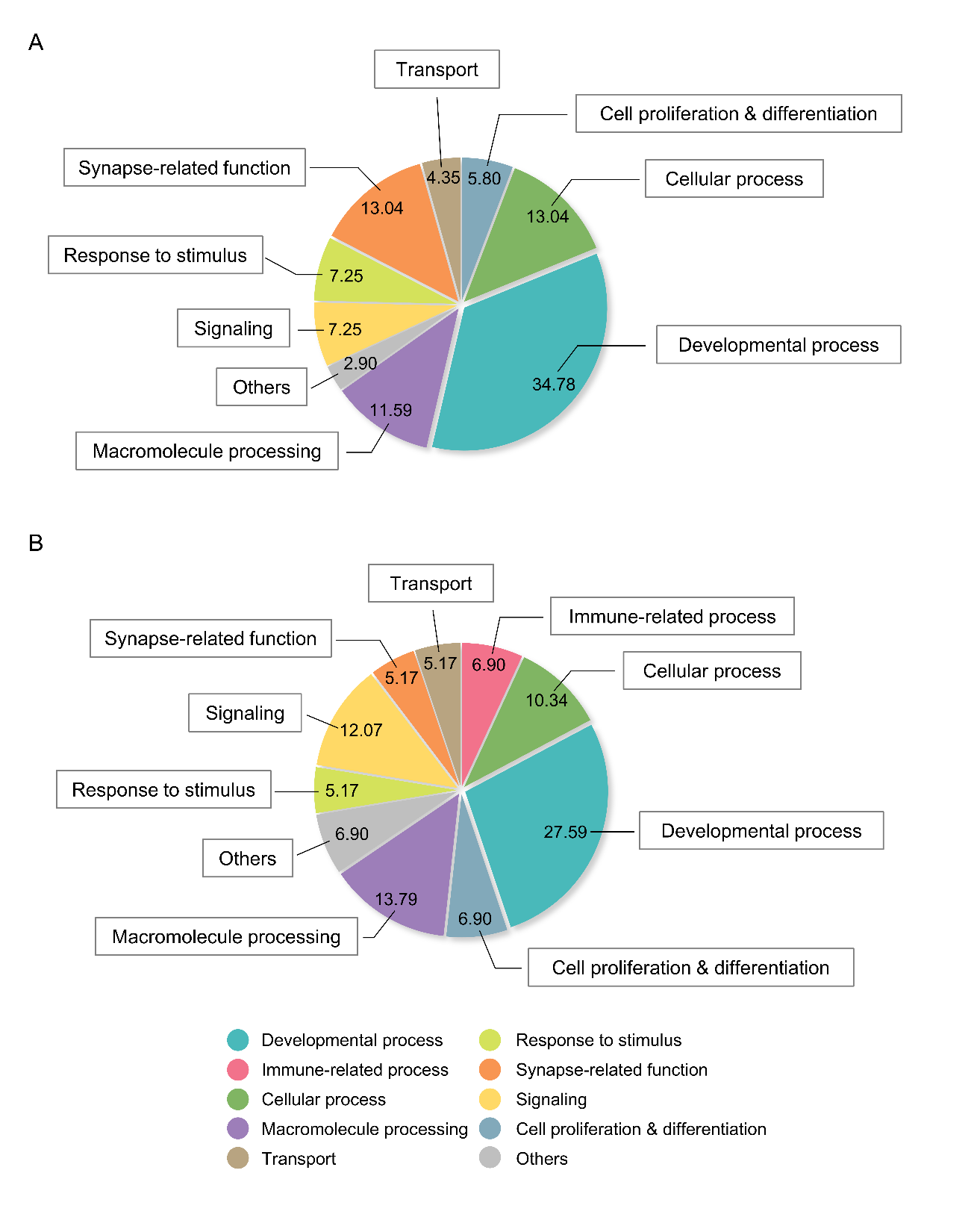
**

**Supplementary Figure S7.** Subcategorization of GO terms in the GO analysis of predicted target genes of DEmiRNAs in the cultured cortical neurons with GATA1 overexpressed. Pie chart showing the subcategorization of GO terms overrepresented in GO analysis of target genes for differentially up-regulated miRNAs **(A)** and down-regulated miRNAs **(B)**. Numbers on pie chart represent the percentage of each category.

**
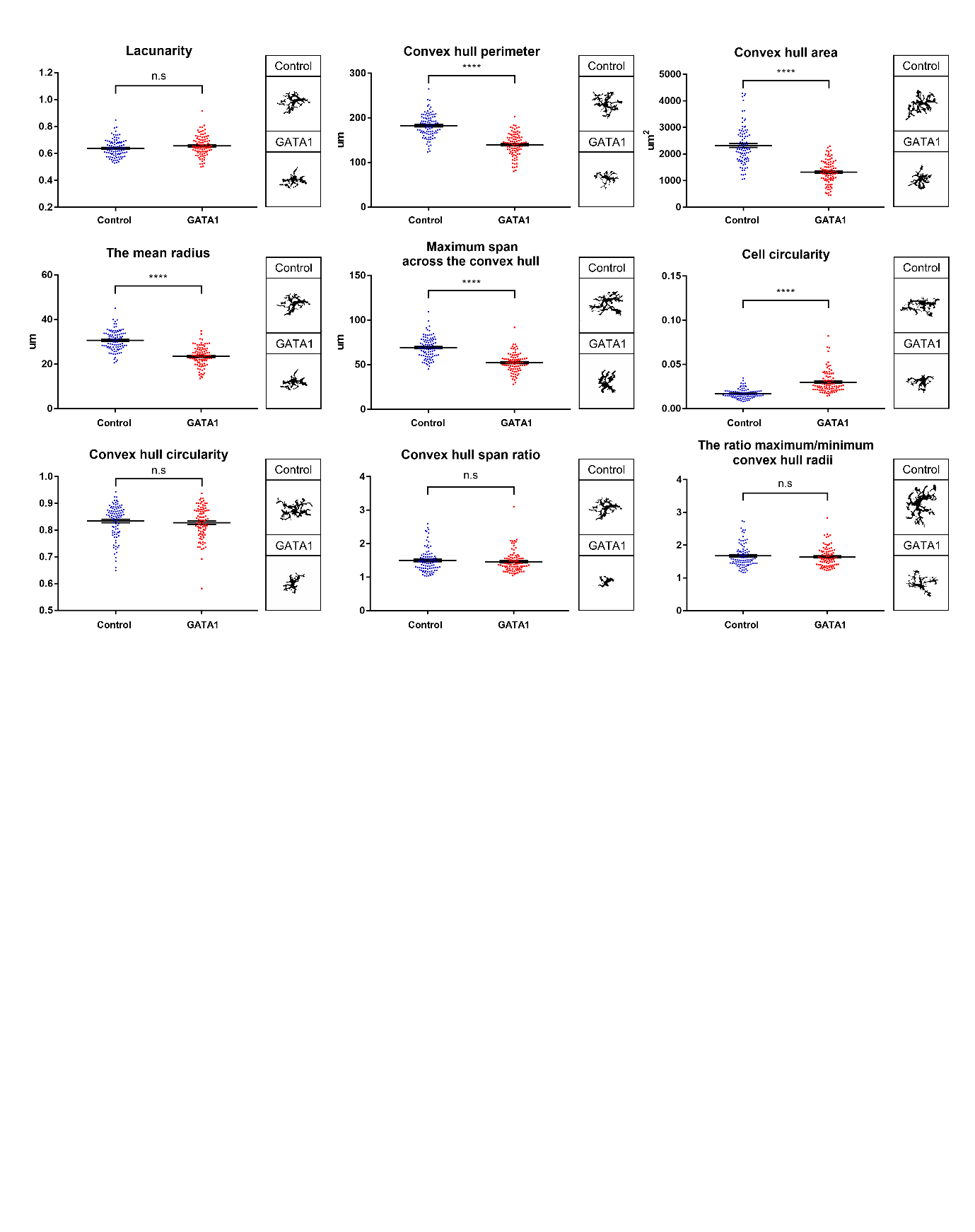
**

**Supplementary Figure S8.** Comparison of morphological parameters. Comparison of morphological parameters between microglial cells in the Control and GATA1 group. Graph shows mean ± SEM of parameters of each cell on Control (n=95) or GATA1 (n=90). Asterisks indicate significant differences (P < 0.0001) by unpaired t-test.

**
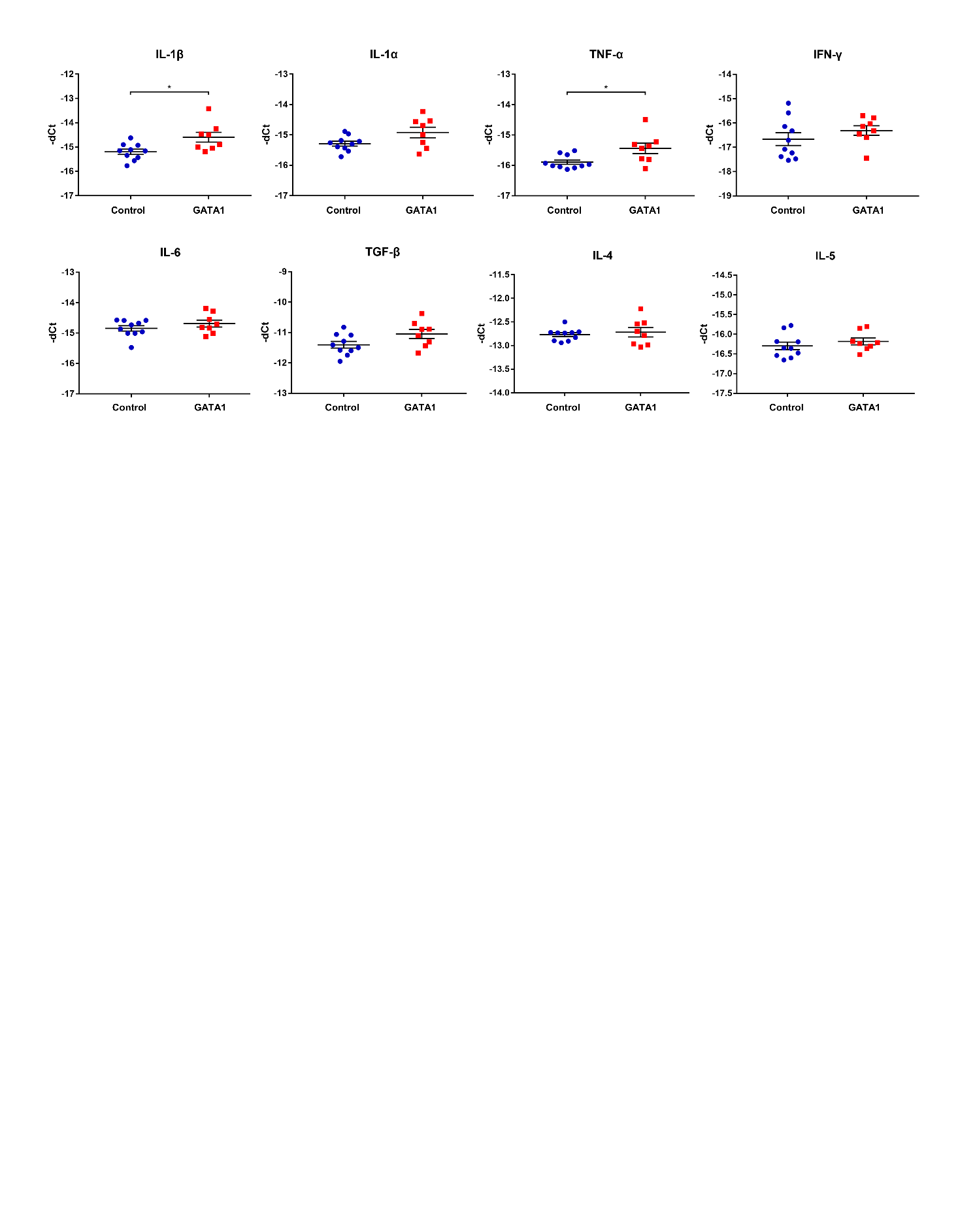
**

**Supplementary Figure S9.** Expression of cytokine genes in the brain with GATA1 overexpressed. The expression patterns of the cytokine genes including pro- and anti-inflammatory cytokine genes were examined in the brain overexpressed with GATA1. Results show that the expression of *IL-1β* and *TNF-α* was significantly increased in the medial prefrontal cortex (mPFC) of GATA1-overexpressed mice. All Ct values were normalized to those of *Gapdh*. Data are shown as mean ± SEM (-dCt) of each gene in control (n=10) and GATA1-overexpressed mice (n=8). Comparative analyses were performed using unpaired t-test (* P < 0.05, ** P < 0.01).

**Supplementary Table Legends**

**Supplementary Table S1.** Summary of sequencing results. CT, Control; GT, GATA1.

**Supplementary Table S2.** Number of ChIPseq peaks of H3K4me3 and H3K27me3. Identification of ChIPseq peak for H3Krme3 and H3K27me3 in the cortical cultured neuron having overexpressed AAV-Control or GATA1. CT, Control; GT, GATA1.

**Supplementary Table S3.** GATA1-specific unique peaks in the promoter region. List of H3K4me3 **(A)** and H3K27me3 **(B)** peaks that are detected only in GATA1-overexpressed group. Peak Score was calculated considering several factors such as normalized tag count, fold change, and clonal fold change. Normalized Tag Count is the number of tags found at the peak, which was normalized to 10 million total mapped tags. Control was input DNA control, which was purified from cells that are cross-linked, fragmented, but without adding any antibody for enrichment.

**Supplementary Table S4.** GO analysis of GATA1-specific unique peak genes in gene promoter region. GATA1-specific unique peak genes of H3K4me3 **(A)** and H3K27me3 **(B)** were used for GO analysis and categorized according to their function. Statistically significant categories were selected (Benjamini < 0.1). BP, biological process; CC, cellular component; MF, molecular function.

**Supplementary Table S5.** DEGs in the cultured cortical neurons with overexpressed GATA1. List of differentially expressed mRNAs in the cultured cortical neurons with overexpressed-GATA1. The P-values were corrected using the Benjamini‒Hochberg method. Genes with raw P-value < 0.05 were considered to be differentially expressed.

**Supplementary Table S6.** GO analysis of DEGs in the cortical neurons with overexpressed GATA1. Up-regulated **(A)** or down-regulated **(B)** genes in the cortical neurons with overexpressed-GATA1 were used for GO analysis and categorized according to their function. Statistically significant categories were selected (P < 0.05). BP, biological process; CC, cellular component; MF, molecular function.

**Supplementary Table S7.** DEmiRNAs in the cultured cortical neurons with overexpressed GATA1. List of DEmiRNAs in the cultured cortical neurons with overexpressed GATA1. The P-values were calculated by Fisher’s exact test and corrected using the Benjamini‒Hochberg method. Genes with raw P-value < 0.05 were considered to be differentially expressed.

**Supplementary Table S8.** List of predicted target genes of DEmiRNAs. The target prediction analysis was performed using five online databases. The genes predicted in two or more databases were selected. The upper table **(A)** is the target list of up-regulated miRNAs and the lower table **(B)** is the target list of down-regulated miRNAs.

**Supplementary Table S9.** GO analysis of predicted target genes of DEmiRNAs in the cortical neurons with overexpressed GATA1. Target genes of up-regulated miRNAs **(A)** or down-regulated miRNAs **(B)** were used for GO analysis and categorized according to their function. Statistically significant categories were selected (Benjamini < 0.1). BP, biological process; CC, cellular component; MF, molecular function.
